# Chicken cathelicidin-3 protected mice from avian pathogenic *Escherichia coli* infection mediated by triggering chemokine CCL3 pathway

**DOI:** 10.1186/s13567-026-01844-5

**Published:** 2026-10-06

**Authors:** Luyao Jiang, Fan Yang, Jiayan Wu, Zhengliang Li, Xiemin Zhu, Hao Pan, Shaotong Zhang, Haochun Pan, Hang Wang, Jingting Yao, Ping Yan, Qingqing Gao, Changchao Huan, Song Gao, Xinan Jiao, Xiufan Liu

**Affiliations:** 1 College of Veterinary Medicine, Yangzhou University, Yangzhou, 225009 China; 2 Jiangsu Co-innovation Center for Prevention and Control of Important Animal Infectious Diseases and Zoonoses, Yangzhou, 225009 China; 3 Key Laboratory of Avian Bioproduct Development, Ministry of Agriculture and Rural Affairs, Yangzhou, 225009 China; 4 Jiangsu Interdisciplinary Center for Zoonoses and Biosafety, Yangzhou University, Yangzhou, 225009 China; 5 Jiangsu Key Laboratory of Zoonosis, Yangzhou University, Yangzhou, 225009 China; 6 Joint International Research Laboratory of Agriculture and Agri-Product Safety, Yangzhou University, Yangzhou, 225009 China

**Keywords:** Host defense peptides, chicken cathelicidin-3, avian pathogenic *Escherichia coli*, mouse, macrophages

## Abstract

Host defense peptides (HDPs) possess both direct bacteriocidal and immunomodulatory activities. In this study, chicken cathelicidin-1 (cCATH-1) and chicken cathelicidin-3 (cCATH-3) were selected based on their markedly different minimum inhibitory concentrations (MICs) against avian pathogenic *Escherichia coli* (APEC) strains, with MICs of cCATH-1 being 4- to 16-fold lower than those of cCATH-3. However, following intraperitoneal challenge with APEC strain E058 (4 × 10⁶ CFU/mouse), 80% of cCATH-3 treated mice survived while 20% of cCATH-1 treated mice survived. Flow cytometry (FC) revealed significantly lower peripheral blood Ly6C^high^ monocytes but higher peritoneal Ly6C^high^ macrophages in cCATH-3-treated mice at 4 and 8 h post-treatment. Peritoneal CCL3 secretion was significantly upregulated in cCATH-3 treated mice at 8 h post-treatment. Upon knockout of macrophages, the protection of cCATH-3 in mice against APEC E058 challenge was markedly abolished. In vitro, peritoneal macrophages treated with cCATH-3 showed stronger bacteriocidal activity than those treated with cCATH-1. Mouse CCL-3 (mCCL3)-specific antibody neutralize the effect of mCCL3 on macrophages chemotaxis in vitro. mCCL3-specific antibody administration resulted in 0% survival in cCATH-3-treated mice versus 60% survival in cCATH-3-treated mice administered with mock antibody, while cCATH-1-treated mice showed 0% survival administered with either mCCL3-specific antibody or mock antibody after APEC E058 strain challenge. In summary, cCATH-3 provides robust protection against APEC infection compared with cCATH-1 in mice, partially through CCL3-mediated macrophage chemotaxis and enhanced bacteriocidal activity in activated macrophages, revealing a novel mechanism of avian cathelicidins and supporting HDPs as alternatives to antibiotics for controlling avian colibacillosis.

**Supplementary Information:**

The online version contains supplementary material available at https://doi.org/10.1186/s13567-026-01844-5.

## Introduction

Since the 1950 s, antimicrobial agents have been widely used in the treatment of avian colibacillosis, followed by the emergence and spread of antimicrobial resistance (AMR) in APEC [1]. With the increasing number of multidrug-resistant pathogens worldwide and the continuous decline in the development efficiency of new antibiotics, there is a need to develop new agents that can not only kill pathogens but also reduce harmful inflammatory responses to replace antibiotics for infection control. Host defense peptides (HDPs) have the potential to fulfil these expectations, and dozens of drugs based on HDPs or their derivatives have entered clinical trial stages, most of which are in phase II and III clinical evaluation, and some have progressed to phase IV clinical stage [2].

HDPs are also known as antimicrobial peptides (AMPs) due to their direct bacteriocidal activity, are now recognized as dual-function molecules that combine direct bacteriocidal and immunomodulatory properties. Their immunomodulatory functions include: (i) recruitment and activation of neutrophils, monocytes, and macrophages [3]; (ii) modulation of pro- and anti-inflammatory cytokine/chemokine networks [3]; (iii) enhancement of phagocytosis and intracellular killing [3]; and (iv) promotion of wound healing and tissue repair [3]. Critically, these immunomodulatory activities may operate independently of direct bacteriocidal effects and can provide protection even when in vitro bacteriocidal activity is weak—a phenomenon central to the present study [3].

The major HDPs in poultry include defensins, cathelicidins, liver-expressed antimicrobial peptide 2 (LEAP2), and NK-lysin [4]. The genomic localization and three-dimensional structures of avian defensins and cathelicidins have been identified [3]. Cationic peptides, including β-defensins and cathelicidins, are capable of killing bacteria by forming pores in the bacterial cell membrane through three distinct mechanisms. One mechanism is the barrel-stave model, in which the cationic peptide is inserted perpendicularly into the membrane; the hydrophobic region interacts with the lipid bilayer, while the hydrophilic region directly contributes to pore formation. The second mechanism is the toroidal model, in which cationic peptides induce inward bending of the membrane, leading to the formation of membrane pores. The third is the carpet model, in which cationic peptides accumulate on the cell membrane surface to a certain threshold to form micelles. When these micelles are removed from the cell membrane, pores are formed. In addition to “punching holes” in bacterial membranes, cationic peptides can also kill bacteria by affecting bacterial DNA and RNA synthesis, protein synthesis, or folding [2, 5, 6]. Notably, the bactericidal kinetics of these peptides are remarkably rapid: avian cathelicidins can kill *Salmonella enteritidis* within 10–30 min [7] and *E. coli* within 30–60 min [8], while chicken NK-lysin can even disrupt the *E. coli* cell membrane within 5 min [9]. This rapid and multifaceted mode of action makes it difficult for bacteria to develop drug resistance to HDPs [10].

Avian cathelicidins, including quail CATH-2, CATH-3, and pheasant CATH-1 shows minimum inhibitory concentrations (MICs) of 1–10 μM for most Gram-positive and Gram-negative bacteria in vitro [10, 11]. These findings demonstrate that HDPs, especially cathelicidins, possess potential bacteriocidal effects under controlled laboratory conditions. However, robust in vitro bacteriocidal activity is not necessarily predictive of in vivo protection, as highlighted by studies showing that some HDPs with weak direct bacteriocidal activity can still confer significant protection through immune cell recruitment and cytokine induction [12, 13].

At present, both bacteriocidal activity and immunomodulatory of avian HDPs against APEC infection have focused primarily on cathelicidins. Direct bacteriocidal activity against APEC is achieved principally through LPS binding and membrane disruption [14–17], which can reduce bacterial load but may not alone ensure host survival. Instead, immunomodulatory mechanisms contribute to disease protection: for example, CATH-B1 downregulates pro-inflammatory cytokines and attenuates excessive macrophage activation, thereby reducing immunopathology and improving survival during APEC infection [16]. Similarly, CATH-2 prevents LPS-induced endotoxic shock by blocking TNF-α and nitric oxide (NO) production, a critical cause of mortality in systemic bacterial infections [17]. The immunomodulatory properties of HDPs are further exemplified by PopuCATH, which exhibits relatively weak direct bacteriocidal activity yet protect the host from *E. coli* challenge by mobilizing neutrophils and monocytes/macrophages to the infection site; stimulating the production of chemokines/cytokines (including CCL-3, CCL-5, and CXCL-1) in macrophages; and enhancing neutrophil phagocytosis through NET release [18]. Collectively, these findings indicate that HDP—mediated protection is not directly related to their bacteriocidal effect, but rather depends on their modulatory nature to trigger host innate immune response.

Therefore, in this study, we compared two avian cathelicidin orthologs, namely chicken CATH-1 (cCATH-1) and chicken CATH-3 (cCATH-3)-which exhibited similar amino acid sequences and structures but markedly different in vitro MICs against APEC E058. Given the growing evidence that in vitro bacteriocidal activity does not reliably predict in vivo protection, we hypothesized that cCATH-3 might confer a robust protection through immunomodulatory mechanisms, particularly macrophage recruitment and activation, despite its weaker direct bacteriocidal activity related to cCATH-1.

## Materials and methods

### APEC E058 strain

Avian pathogenic *Escherichia coli* (APEC) E058 (O2 serogroup) is a highly virulent strain isolated from a diseased chicken with colibacillosis in our laboratory in the 1990s. Its virulence characteristics, genomic features, and pathogenic mechanisms have been extensively characterized in our previous studies [19–25]. In this study, the bacteria were cultured in Mueller Hinton II Broth (Qingdao Rishui Bio·Technologies Co., Ltd, Qingdao, Shandong, China, Cat#: 11865) at 37 °C with shaking at 220 rpm for 10–12 h or on Luria–Bertani (LB) agar plates (1% Tryptone (Oxoid, Cat#: LP0042B), 0.5% Yeast Extract (Oxoid, Cat#: LP0021B), 1% NaCl (Sinopharm, Cat#: 10019318), and 1.5% Agar (Gentihold, Cat#: A1101)) at 37 °C for an appropriate period.

### Animals and ethics for animal experiments

C57BL/6J mice were purchased from the Comparative Medical Center of Yangzhou University (Yangzhou, Jiangsu, China, SCXK(Su)2022-0009). Specific pathogen-free (SPF) chickens were purchased from Beijing Boehringer Ingelheim Viton Biotechnology Co., LTD (Beijing, China, SCXK(Jing)2019–0007). All experiments were performed in accordance with the animal ethics guidelines approved by the Institutional Animal Care and Ethics Committee of Yangzhou University.

### Antimicrobial peptide synthesis

Antimicrobial peptides were synthesized by GL Biochem (Shanghai) Ltd. (Shanghai, China) using Fmoc-based solid-phase synthesis and were purified by reverse-phase column chromatography HPLC, with a purity exceeding 95%. Quality control was confirmed by mass spectrometry (cCATH-1: observed 3338.09 Da, expected 3338.14 Da; cCATH-3: observed 3547.71 Da, expected 3547.34 Da) and analytical HPLC (cCATH-1 purity: 96.89%; cCATH-3 purity: 96.82%).

### Determination of the minimum inhibitory concentration (MIC) of synthesized cCATH-1 and cCATH-3

After dissolving cCATH-1/cCATH-3 in PBS (10 mM, pH 7.3), the peptides were serially diluted and added to a sterile 96-well plate with 50 μL per well, and a well without antimicrobial peptides was used as a naive bacterial growth control.

*Inoculum preparation* After 10–12 h incubation in MHB II broth medium, the APEC E058 strain inoculum was diluted to 3.3 × 10^5^ colony forming units (CFU)/mL with fresh MHB II broth medium. 150 μL of bacterial suspension was added to each well to achieve a final bacterial concentration of 5 × 10^4^ CFU per well. After the 96-well plates were lidded, the cells were incubated at 37 °C for 18–24 h. The MIC was determined as the lowest drug concentration that completely inhibited bacterial growth in the 96-well plate [26, 27].

### Determination of hemolytic concentration (HC_50_) of synthesized cCATH-1 and cCATH-3

Fresh blood (1 mL) was drawn from each of five 8-week-old SPF chickens(Beijing Boehringer Ingelheim Viton Biotechnology Co., Ltd., Beijing, China), combined to yield a 5 mL pooled blood sample and placed into a biochemical tube containing heparin, mixed, centrifuged at 3000 × *g* for 10 min at 4 °C, and the supernatant was discarded. Erythrocytes were washed with PBS (10 mM, pH 7.3) and centrifuged at 3000 × *g* for 10 min at 4 °C, the supernatant was discarded, and the process was repeated three times. The erythrocytes were then diluted in PBS to a concentration of 4% (v/v). The peptide was diluted with PBS to obtain final concentrations of 1, 0.25, 0.0625, 0.0156, and 0.0039 mg/mL, respectively. 100 μL of 4% erythrocytes were mixed with 100 μL of the above diluted peptides in a well of a 96-well plate and incubated at 37 °C for 1 h. The mixture was then centrifuged at 2000 × *g* for 10 min, and 100 μL of the supernatant was collected to measure the absorbance (OD) at 450 nm using a microplate reader. PBS was used as a negative control, and 0.1% Triton X-100 (Sigma-Aldrich, Taufkirchen, Germany, Cat#: X100) (diluted in PBS) was used as a positive control. The percentage of hemolysis was calculated according to the formula: hemolysis (%) = $\frac{\mathrm{A}\mathrm{p}-\mathrm{A}\mathrm{o}}{\mathrm{A}100-\mathrm{A}\mathrm{o}}$ × 100% (Ap: absorbance value of experimental group, Ao: absorbance value of negative control, A100: absorbance value of the positive control.), and the concentration corresponding to 50% hemolysis rate was calculated using SPSS software as HC50 [28].

### Ultrastructure observation of APEC E058 treated with or without cathelicidins under a transmission electron microscope (TEM)

APEC E058 was cultured in MHB II broth overnight at 37 °C with shaking at 220 rpm, and then inoculated into fresh MHB II broth at a 1% (v/v) ratio and incubated under the same conditions for 3–4 h until the logarithmic growth phase. The bacteria collected by centrifugation at 1000 × *g* were resuspended in PBS and washed three times, then adjusted to an OD_600nm_ of 0.2 using PBS. cCATH-1/cCATH-3 diluted in PBS was mixed with the bacterial suspension to a final concentration of 1 × MIC. After incubation at 37 °C for 1 h, bacteria were collected by centrifugation at 5000 × *g* and washed three times with PBS [28]. Bacteria were resuspended in electron microscope fixative (Wuhan ServiceBio Technology Co., Ltd., Wuhan, Hubei, China, Cat#: G1102), stored in the dark at 4 °C, and fixed overnight for ultrathin section preparation. Embedding, sectioning, staining, and observation were performed at Wuhan ServiceBio Technology Co., Ltd (Wuhan, Hubei, China).

### Prophylactic efficacy of cCATH-1 and cCATH-3 in a mouse model of APEC E058 infection

Twenty C57BL/6J mice (female, 6–8 weeks old) were randomly assigned into four groups, with five mice in each group. Four hours before challenge, the treatments were administered as follows: cCATH-1 or cCATH-3 (10 mg/kg body weight) dissolved in 200 μL PBS (10 mM, pH 7.3) was intraperitoneally injected into mice in the corresponding groups, whereas mice in the challenge control group and the mock group received an equal volume of PBS. Subsequently, mice in the cCATH-1 or cCATH-3 treatment groups and the challenge control group were intraperitoneally inoculated with APEC E058 strain (cultured to the logarithmic phase on LB agar plates, 4 × 10^6^ CFU/mouse suspended in 200 μL PBS), while mice in the mock group were intraperitoneally injected with the same volume of PBS. The mortality (survival rate) of mice in each group was recorded for 7 days after challenge to evaluate the shelter of cCATH-1/cCATH-3 against APEC E058 strain challenge in mice [18, 29].

### Differential cell populations sorted by flow cytometry (FC)

Fifteen C57BL/6J mice (female, 6–8 weeks old) were randomly divided into 3 groups with 5 mice in each group. Mice in two groups were intraperitoneally injected with cCATH-1 or cCATH-3 (10 mg/kg body weight) dissolved in 200 μL PBS, respectively, while mice in the mock group were injected with the same volume of PBS via the same route.

The peritoneal lavage cells and peripheral blood cells were collected at 4 h and 8 h after cathelicidin or PBS administration for chemotactic kinetics determination [18, 30]. Collected cells were washed with PBS containing 2% fetal bovine serum (FBS, Lonsera, Suzhou ShuangRu Biotechnology Co., Ltd., Suzhou, Jiangsu, China, Cat#: S711-001S) and incubated with anti-FcγR blocking antibody (BD Biosciences, Clone: 2.4G2, New Jersey, USA, Cat#: 553141, FC (1: 100), diluted in PBS containing 0.5% BSA (Solarbio, Beijing, China, Cat#: A8020). After incubation at 4 °C for 30 min, cells were washed with 0.5% BSA in PBS three times by centrifugation and then resuspended in 100 μL of 0.5% BSA in PBS.

For myeloid cell analysis, cells were stained with APC-Cy7/anti-CD45 (BioLegend, Clone: 30-F11, Cat#: 103116, San Diego, California, USA, FC (1: 100)), PE/anti-CD11b (BioLegend, Clone: M1/70, Cat#: 101207, FC (1: 100)), PE/Cy7/anti-Ly6G (BioLegend, Clone: 1A8, Cat#: 127618, FC (1: 100)), FITC/anti-Ly6C (BD Biosciences, Clone: AL-21, Cat#: 553104, FC (1: 100)), and APC/F4/80 (BioLegend, Clone: BM8, Cat#: 123116, FC (1: 100)) at 4 °C for 30 min. Cells were washed with PBS three times by centrifugation and then re-suspended in 200 μL PBS. Stained cells were analyzed using a CytoFLEX S flow cytometer (Becton, Dickinson and Company, New Jersey, USA) with FlowJo V10 software [18, 30].

### Mouse peritoneal macrophage preparation

Preparation of mouse peritoneal macrophages: C57BL/6J mice (6–8 weeks old) were acclimated in an individual ventilated cage (IVC) isolator system (Suzhou Fengshi Laboratory Animal Equipment Co., Ltd., China) with HEPA-filtered air supply and 12:12 h light–dark cycle for 1 week before use. On days 1–3, 1 mL of 4% sodium thioglycolate medium (Sigma-Aldrich, Taufkirchen, Germany, Cat#: B2551) was intraperitoneally injected into the mice once daily. On day 4, the mice were euthanized using isoflurane (Aladdin, Cat#: C153359, Shanghai, China), and the peritoneal cavity was lavaged with RPMI 1640 medium (HyClone, Logan, Utah, USA, Cat#: SH30027.01) containing 100 U/mL penicillin, 100 μg/mL streptomycin, and 2.5 μg/mL amphotericin B (Sangon Biotech, Shanghai, China, Cat#: B540733), supplemented with 4% FBS. The lavage fluid was collected and centrifuged at 300 × *g* for 10 min at 4 °C, and the harvested cells were washed three times with RPMI 1640 medium (300 × *g*, 5 min each time, 4 °C), then adjusted to a concentration of 3 × 10^5^ cells/mL. The cell suspension was seeded into 12-well plates at 1 mL/well and allowed to adhere to the well surface. After 8–12 h of incubation, non-adherent cells were removed by washing with the same medium. After digestion using 0.25% trypsin–EDTA (New Cell & Molecular Biotech Co., Ltd, Suzhou, Jiangsu, China, Cat#: C100C1), the cells were washed in RPMI 1640 medium with 4% FBS and prepared for use [18, 29, 31].

### Cytotoxicity of cCATH-1 and cCATH-3 on mouse peritoneal macrophages determined by CCK-8 measure

About 5 × 10^4^ mouse peritoneal macrophages per well (100 μL) were seeded in 96-well plates, a blank control (medium only, no cells) and a cell control (cells in medium, no peptides) were included in each plate. After cell adhesion, cCATH-1 and cCATH-3 at different concentrations diluted in RPMI 1640 medium (100 U/mL penicillin, 100 μg/mL streptomycin, and 2.5 μg/mL amphotericin B) containing 4% FBS were added. After 4 h, the medium was discarded, and CCK-8 working solution was prepared by diluting CCK-8 reagent (Beyotime, Shanghai, China, Cat#: C0042) 1:10 in pre-warmed RPMI 1640 medium (without FBS or antibiotics), and 100 μL was added to each well. The plate was incubated for 1 h at 37 °C in 5% CO_2_ under the dark. Absorbance was measured at 450nm using a BIO-RAD iMark microplate reader (Bio-Rad Laboratories, California, USA), with a reference wavelength of 630nm to correct for non-specific background. The plate was gently shaken for 1 min before reading to ensure uniform color distribution. Blank control wells (medium only) were used to subtract background absorbance [32, 33].

Cell viability (%) was calculated using the following formula: Cell viability (%)=$\frac{Asample-Ablank}{Acell control-Ablank}\times 100\%$. Where A_sample_ is the absorbance of peptide-treated wells, A_blank_ is the absorbance of medium-only wells, and A_cell control_ is the absorbance of untreated cell wells.

The 50% cytotoxic concentration (CC₅₀) was calculated by non-linear regression analysis of the dose–response curve using GraphPad Prism 8 (GraphPad Software, USA). Experiments were performed in triplicate on three independent occasions.

### Phagocytosis of APEC E058 strain by mouse peritoneal macrophages in the presence of cCATH-1 or cCATH-3

In this experiment, 5 × 10^5^ mouse peritoneal macrophages in 1 mL RPMI 1640 medium with 4% FBS (without antibiotics) were preincubated with cCATH-1 or cCATH-3 (final concentration of 10 μM) dissolved in the same RPMI 1640 medium in a 12-well plate at 37 °C and 5% CO_2_ for 2 h [21, 34]. After washing three times with the same RPMI 1640 medium, 1 mL of fresh RPMI 1640 medium was added to each well until use.

#### Phagocytosis of inactivated APEC E058 strain by mouse peritoneal macrophages

APEC E058 strain was grown overnight in MHB II broth at 37 °C with shaking at 220 rpm, then 1% (v/v) inoculum was transferred into fresh MHB II broth and incubated for 3–4 h to reach the logarithmic growth phase. After washing with PBS, bacteria were resuspended in diluted CFDA SE dye (Beyotime, Shanghai, China, Cat#: C1031) (diluted to 10 μM in PBS) and incubated at 37 °C for 30 min for fluorescent staining.

The stained bacteria were incubated with 1% paraformaldehyde (Sangon, Shanghai, China, Cat#: A500684) dissolved in PBS at 37 °C for 1 h to inactivate them and then washed five times with PBS. The fluorescently stained APEC E058 strain was adjusted to 5 × 10^9^ CFU/mL at a multiplicity of infection (MOI) of 100 (following a 5-fold dilution, where an OD_600nm_ of 1 was achieved, then 10 μL PBS-suspended bacteria and 90 μL RPMI 1640 medium with 4% FBS without antibiotics were added to the well, resulting in 1.1 mL total volume with about 5 × 10^7^ CFU bacteria/well), and incubated with mouse peritoneal macrophages obtained in the section “Mouse peritoneal macrophage preparation” for 0.5, 2, and 4 h at 37 °C in a 5% CO_2_ incubator. Subsequently, the cells were digested using trypsin and washed three times with PBS (1000 × *g*, 4 °C, 10 min), and extracellular fluorescence was quenched by trypan blue (BBI, Shanghai, China, Cat#: A601140, 15 mg/mL prepared in PBS). For quenching of extracellular fluorescence, trypan blue solution was added to each well at a ratio of 1:10 (v/v), and the culture was immediately collected followed by centrifugation. After three washes with PBS, the cells were resuspended for fluorescence measurement. Carboxyfluorescein diacetate succinimidyl ester (CFSE) fluorescence was analyzed by flow cytometry to evaluate the phagocytic capacity of mouse peritoneal macrophages toward inactivated APEC E058 strain [18].

#### Intracellular survival assay of live APEC E058 strain in mouse peritoneal macrophages

APEC E058 strain was grown overnight in MHB II broth at 37 °C with shaking at 220 rpm, then 1% (v/v) inoculum was transferred into fresh MHB II broth and incubated for 3–4 h to the logarithmic growth phase. The bacteria were harvested by centrifugation at 3000 × *g* (4 °C, 10 min) and washed three times with PBS (10 mM, pH 7.3). Pelleted APEC E058 bacterial cells were diluted to 1 × 10^9^ CFU/mL in PBS and then diluted to 5 × 10^7^ CFU/mL in the above RPMI 1640 medium. In each well, 5 × 10^7^ CFU of E058 strain was mixed with peritoneal macrophages to achieve an MOI of 100. After 1 h of incubation, the cells were washed three times with RPMI 1640 medium. Subsequently, 1 mL of RPMI 1640 medium containing 100 μg/mL gentamicin (Sangon Biotech, Shanghai, China, Cat#: A620217) was added to each well and incubated for 1.5 h to eliminate extracellular bacteria. The supernatant was then discarded, and the cells were washed three times with RPMI 1640 medium, followed by the addition of 0.5 mL RPMI 1640 medium supplemented with 4% FBS, and this time point was defined as 0 h. At 0.5, 1, 2, 4, 6, 8, and 12 h, the cells were washed three times with PBS and lysed using 0.1% Triton X-100 to release intracellular bacteria. The number of intracellular bacteria was determined by plate counting [21, 34].

### Chemotactic effect of cCATH-3 on mouse peritoneal macrophages

In a separate culture system, macrophages were prepared in RPMI 1640 medium supplemented with 4% FBS at a concentration of 5 × 10^6^ cells/mL. In 24-well plates, 100 μL of macrophage suspension was added to the upper chamber of a Transwell insert (5.0 μm) (Guangzhou JET Bio-filtration Co., Ltd., Guangzhou, Guangdong, China, TCS004024). Subsequently, 500 μL of RPMI 1640 medium with 4% FBS containing 10 μM cCATH-1 or cCATH-3 was placed in the lower chamber. The 24-well plates assembled with the Transwell system were incubated at 37 °C in a 5% CO_2_ incubator for 8 h. Macrophages that migrated to the lower chamber were counted using a hemocytometer, and the increase in cell number in the lower chamber was considered as migrated cells [18, 29].

For the co-culture system, macrophages were resuspended in RPMI 1640 medium containing 4% FBS at a concentration of 5 × 10^6^ cells/mL. Macrophages were initially seeded into 24-well plates at 500 μL per well. After adherence of macrophages, the culture supernatant was replaced with 500 μL of RPMI 1640 medium with 4% FBS containing 10 μM cCATH-1 or cCATH-3 in the lower chamber. Then, 100 μL of macrophage suspension was added to the upper chamber of a Transwell insert (5.0 μm). The plates assembled with the Transwell system were incubated at 37 °C in a 5% CO_2_ incubator for 8 h. Macrophages remaining in the upper chamber were counted using a hemocytometer, and the decrease in macrophage number in the upper chamber was defined as migrated cells [18, 29].

### Validation of the protection of cCATH-3 in macrophage knockout mice against APEC E058 strain challenge

Forty C57BL/6J mice (female, 6–8 weeks old) were randomly divided into 8 groups with 5 mice in each group. Mice in groups 1 and 2 were intraperitoneally administered anti-CSF1R antibody (BioXcell, Clone: AFS98, West Lebanon, New Hampshire, USA, Cat#: BE0213), whereas mice in groups 3 and 4 were intraperitoneally treated with IgG2a antibody (BioXcell, Clone: 2A3, Cat#: BE0089), and mice in groups 5 and 6 received PBS (10 mM, pH 7.3, 200 μL/mouse). Mice in groups 1–4 were intraperitoneally injected with 1 mg of the corresponding antibody diluted in 200 μL PBS per mouse on day 0, followed by administration of 0.3 mg antibody per mouse on day 1 and day 2, respectively [18, 29]. The challenge-only group (group 7) was injected with the same volume of PBS via the same route and at same time points. Mice in mock group (group 8) were left untreated (Table 1).

**Table 1:** **CSF1R antibody and/or cCATHs treatment followed by APEC E058 intraperitoneal challenge in C57BL/6 J mice**

Group No	Antibody treatment (i.p. injection)	cCATHs treatment (i.p. injection) on Day 3	Infection treatment (i.p. injection)	Notes
1	Anti-CSF1R antibody (1 mg on day 0, 0.3 mg on days 1 and 2)	cCATH-1 (10 mg/kg body weight)	4 × 10^6^ CFU APEC E058 strain (peritoneal lavage collected at 18 h post-challenge)	Challenge-only group
2		cCATH-3 (10 mg/kg body weight)		
3	IgG2a antibody (1 mg on day 0, 0.3 mg on days 1 and 2)	cCATH-1 (10 mg/kg body weight)		
4		cCATH-3 (10 mg/kg body weight)		
5	PBS (200 μL on days 0, 1 and 2)	cCATH-1 (10 mg/kg body weight)		
6		cCATH-3 (10 mg/kg body weight)		
7		PBS (200 μL)		
8	Untreated	Untreated	Untreated	Mock group

On day 3, cCATH-1 (10 mg/kg body weight) was intraperitoneally administered to mice in groups 1, 3, and 5, while the same dose of cCATH-3 was intraperitoneally administered to mice in groups 2, 4, and 6. Mice in group 7 were intraperitoneally given an equal volume of PBS, whereas mice in group 8 received no treatment. Four hours after administration of cathelicidins, each mouse in groups 1–7 was intraperitoneally challenged with 4 × 10^6^ CFU of APEC E058 strain, and peritoneal lavage fluid was collected at 18 h post-challenge for bacterial enumeration [18, 29].

### Determination of cytokines in cathelicidin-treated murine peritoneal macrophages with or without APEC E058 strain challenge

Mouse peritoneal macrophages were seeded at a density of 2 × 10^5^ cells/well in a 24-well cell culture plate (RPMI 1640 with 4% FBS, without antibiotics) and allowed to adhere. APEC E058 bacterial cells were prepared as described in the section “Phagocytosis of live APEC E058 strain by mouse peritoneal macrophages”.

After cell adhesion, suspended cells were thoroughly washed using RPMI 1640 supplemented with 4% FBS. The experimental trials were arranged as indicated in Table 2. Subsequently, cells in groups 1–6 were exposed to different final concentrations (5 μM, 7.5 μM, and 10 μM) of cCATH-1 or cCATH-3 for 4 h, after which the culture supernatant was collected for ELISA analysis to measure the secretion levels of CXCL-1, CXCL-2, CCL-3, CCL-4, and CCL-5, following the manufacturer’s instructions (ml063493 for CCL-3, ml058594 for CCL-4, ml057800 for CCL-5, ml038369 for CXCL-1, ml058180 for CXCL-2, Shanghai Enzyme-linked Biotechnology Co., Ltd., Shanghai, China). The cells were washed three times with PBS, then lysed with 500 μL of FreeZol Reagent to disrupt cellular structures, and the lysate was collected and stored at – 70 °C for RNA extraction. The transcription levels of Actb, IFN-β, IFN-γ, TNF-α, CCL-1, CCL-2, CCL-3, CCL-4, CCL-5, CCL-6, CCL-7, CCL-8, CCL-9, CCL-11, CCL-17, CCL-22, CCL-24, CXCL-1, CXCL-2, CXCL-3, CXCL-5, CXCL-7, CXCL-9, CXCL-10, CXCL-11, CXCL-14, CSF-1, CSF-2, IL-1β, IL-4, IL-6, IL-10, IL-12, IL-15, IL-17a, IL-23, and IL-1RN were determined by real-time quantitative PCR (qPCR) (primers for qPCR listed in Table 3) [18, 35–38]. Cells in groups 7–8 were treated with cCATH-1 or cCATH-3 for 4 h, followed by washing to remove cathelicidins. Then, 0.5 mL of APEC E058 bacterial cells suspended in RPMI 1640 with 4% FBS (1 × 10^6^ CFU/mL) were added and incubated for another 4 h. The secretion levels of CXCL-1, CXCL-2, CCL-3, CCL-4, and CCL-5 in cell culture supernatant were determined using the commercial ELISA kits mentioned above, and their transcriptional levels in cell lysates were determined by qPCR as previously described [18, 29, 31]. Cells in groups 9–10 were first incubated with 0.5 mL of APEC E058 bacterial cells suspended in RPMI 1640 with 4% FBS (1 × 10^6^ CFU/mL) for 4 h, then washed and further incubated with RPMI 1640 containing 4% FBS, 0.5 mg/mL gentamicin, and 10 μM cCATH-1 or cCATH-3 for another 4 h, and subsequent procedures were the same as those in groups 7–8. Cells in groups 11–12 were incubated with 0.5 mL of both APEC E058 bacterial cells (1 × 10^6^ CFU/mL) and 10 μM cCATH-1 or cCATH-3 for 4 h in RPMI 1640 with 4% FBS, and subsequent procedures were the same as those in groups 7–8. Cells in group 13 were incubated with 0.5 mL of APEC E058 culture suspended in RPMI 1640 with 4% FBS (1 × 10^6^ CFU/mL) for 4 h, and subsequent procedures were the same as those in groups 7–8. Cells in group 14 were left untreated as a negative control and cultured until the end of the experiment. Treatment groups for cytokine determination in murine peritoneal macrophages treated with cathelicidins and/or APEC E058 strain are summarized in Table 2. Each group had three replicates [16, 39].

**Table 2:** **Determination of cytokines in cathelicidin-treated murine peritoneal macrophages with or without APEC E058 strain challenge**

Group No	Cathelicidins	Final concentration of cathelicidins (μM)	Incubation time for cathelicidins (hours)	APEC E058 strain	CFUs of APEC E058 strain in each well	Incubation time for APEC E058 strain (hours)
1	cCATH-1	5	4	–	–	–
2	cCATH-1	7.5	4	–	–	–
3	cCATH-1	10	4	–	–	–
4	cCATH-3	5	4	–	–	–
5	cCATH-3	7.5	4	–	–	–
6	cCATH-3	10	4	–	–	–
7	cCATH-1	10	4	+	1 × 10^6^ CFU/mL	4
8	cCATH-3	10	4	+		4
9	cCATH-1	10	4	+		4
10	cCATH-3	10	4	+		4
11	cCATH-1	10	4	+		4
12	cCATH-3	10	4	+		4
13	–	–	–	+		4
14	–	–	–	–	–	–

“+” means involved, and “–” means not involved.

**Table 3:** **Primers used in real-time quantitative PCR for cytokine determination**

Gene ID	Primer names	Primer sequence	References
11461	M-Actb-F	AACAGTCCGCCTAGAAGCAC	[18, 35–38]
	M-Actb-R	CGTTGACATCCGTAAAGACC	[18, 35–38]
15977	M-IFN-β-F	AGCTCCAAGAAAGGACGAACA	[35–38]
	M-IFN-β-R	GGCAGTGTAACTCTTCTGCAT	[35–38]
15978	M-IFN-γ-F	ACAGCAAGGCGAAAAAGGATG	[35–38]
	M-IFN-γ-R	TGGTGGACCACTCGGATGA	[35–38]
21926	M-TNF-α-F	CGGTGCCTATGTCTCAGCCT	[18, 35–38]
	M-TNF-α-R	GAGGGTCTGGGCCATAGAAC	[18, 35–38]
20290	M-CCL-1-F	GCAAGAGCATGCTTACGGTCT	[18, 35–38]
	M-CCL-1-R	TAGTTGAGGCGCAGCTTTCT	[18, 35–38]
20296	M-CCL-2-F	GCTGTAGTTTTTGTCACCAAGC	[18, 35–38]
	M-CCL-2-R	GTGCTGAAGACCTTAGGGCA	[18, 35–38]
20302	M-CCL-3-F	CCATATGGAGCTGACACCCC	[18, 35–38]
	M-CCL-3-R	TCAGGAAAATGACACCTGGCT	[18, 35–38]
20303	M-CCL-4-F	CCAGCTGTGGTATTCCTGACC	[18, 35–38]
	M-CCL-4-R	AGCAAGGACGCTTCTCAGTG	[18, 35–38]
20304	M-CCL-5-F	GACAGCACATGCATCTCCCA	[18, 35–38]
	M-CCL-5-R	GTGTCCGAGCCATATGGTGA	[18, 35–38]
20305	M-CCL-6-F	TCAAGCCGGGCATCATCTTT	[18, 35–38]
	M-CCL-6-R	CTGCCCTCCTTCTCAAGCAA	[18, 35–38]
20306	M-CCL-7-F	GTCTGCCAGCTCTCACTGAA	[18, 35–38]
	M-CCL-7-R	GCATTGGGCCCATCTGGTTG	[18, 35–38]
20307	M-CCL-8-F	GACGCTAGCCTTCACTCCAA	[18, 35–38]
	M-CCL-8-R	GAGCCTTATCTGGCCCAGTC	[18, 35–38]
20308	M-CCL-9-F	CAGGCCGGGCATCATCTTTA	[18, 35–38]
	M-CCL-9-R	AGTAGCTGGCAGTTCACACC	[18, 35–38]
20292	M-CCL-11-F	GAATCACCAACAACAGATGCAC	[35–38]
	M-CCL-11-R	ATCCTGGACCCACTTCTTCTT	[35–38]
20295	M-CCL-17-F	GACGACAGAAGGGTACGGC	[35–38]
	M-CCL-17-R	GCATCTGAAGTGACCTCATGGTA	[35–38]
20299	M-CCL-22-F	AGGTCCCTATGGTGCCAATGT	[35–38]
	M-CCL-22-R	CGGCAGGATTTTGAGGTCCA	[35–38]
56221	M-CCL-24-F	ATTCTGTGACCATCCCCTCAT	[35–38]
	M-CCL-24-R	TGTATGTGCCTCTGAACCCAC	[35–38]
14825	M-CXCL-1-F	ACCGAAGTCATAGCCACACTC	[18, 35–38]
	M-CXCL-1-R	CTCCGTTACTTGGGGACACC	[18, 35–38]
20310	M-CXCL-2-F	CCAGACAGAAGTCATAGCCACT	[18, 35–38]
	M-CXCL-2-R	GGTTCTTCCGTTGAGGGACA	[18, 35–38]
330122	M-CXCL-3-F	CCCAGACAGAAGTCATAGCCA	[18]
	M-CXCL-3-R	GTGAGGGGCTTCCTCCTTTC	[18]
20311	M-CXCL-5-F	GCCCCTTCCTCAGTCATAGC	[18, 35–38]
	M-CXCL-5-R	AGCTTTCTTTTTGTCACTGCCC	[18, 35–38]
57349	M-CXCL-7-F	TGGGCTTCAGACTCAGACCT	[18, 35–38]
	M-CXCL-7-R	ATGGGTCCATGCCATCAGATTT	[18, 35–38]
17329	M-CXCL-9-F	GGAGTTCGAGGAACCCTAGTG	[35–38]
	M-CXCL-9-R	GGGATTTGTAGTGGATCGTGC	[35–38]
15945	M-CXCL-10-F	CCACGTGTTGAGATCATTGCC	[18, 35–38]
	M-CXCL-10-R	GAGGCTCTCTGCTGTCCATC	[18, 35–38]
56066	M-CXCL-11-F	CCACAGCTGCTCAAGGCTTC	[18, 35–38]
	M-CXCL-11-R	AACTTTGTCGCAGCCGTTAC	[18, 35–38]
57266	M-CXCL-14-F	GTGGACGGGTCCAAGTGTAA	[18, 35–38]
	M-CXCL-14-R	CCTCGCAGTGTGGGTACTTT	[18, 35–38]
12977	M-CSF-1-F	CTCTAGCCGAGGCCATGTG	[18, 35–38]
	M-CSF-1-R	CGCCCCACAGAAGAATCCAA	[18, 35–38]
12981	M-CSF-2-F	CAGGGTCTACGGGGCAATTT	[18, 35–38]
	M-CSF-2-R	CACAGTCCGTTTCCGGAGTT	[18, 35–38]
16176	M-IL-1β-F	GAAATGCCACCTTTTGACAGTG	[18, 35–38]
	M-IL-1β-R	TGGATGCTCTCATCAGGACAG	[18, 35–38]
16189	M-IL-4-F	AAAATCACTTGAGAGAGATCATCGG	[18, 35–38]
	M-IL-4-R	GTTGCTGTGAGGACGTTTGG	[18, 35–38]
16193	M-IL-6-F	AGTTGCCTTCTTGGGACTGA	[18, 35–38]
	M-IL-6-R	TCCACGATTTCCCAGAGAAC	[18, 35–38]
16153	M-IL-10-F	GGGGCCAGTACAGCCGGGAA	[18, 35–38]
	M-IL-10-R	CTGGCTGAAGGCAGTCCGCA	[18, 35–38]
16183	M-IL-12-F	TGGTTTGCCATCGTTTTGCTG	[18, 35–38]
	M-IL-12-R	ACAGGTGAGGTTCACTGTTTCT	[35–38]
16168	M-IL-15-F	CATCCATCTCGTGCTACTTGTG	[35–38]
	M-IL-15-R	GCCTCTGTTTTAGGGAGACCT	[18, 35–38]
16171	M-IL-17a-F	TTTAACTCCCTTGGCGCAAAA	[18, 35–38]
	M-IL-17a-R	CTTTCCCTCCGCATTGACAC	[18, 35–38]
83430	M-IL-23-F	CAGCAGCTCTCTCGGAATCTC	[35–38]
	M-IL-23-R	TGGATACGGGGCACATTATTTTT	[35–38]
16181	M-IL-1RN-F	GCGGACGATCACTCCTTCTG	[18, 35–38]
	M-IL-1RN-R	AGCCCCACATATTTGAAATTCCA	[18, 35–38]

### Real-time quantitative PCR for cytokine determination

RNA was extracted from collected cell lysates using FreeZol Reagent according to the manufacturer’s instructions (Vazyme, Nanjing, Jiangsu, China, R711-02). Reverse transcription was performed using HiScript II Q RT SuperMix for qPCR (+ gDNA wiper) (Vazyme, R223-01). Quantitative PCR was carried out using AceQ Universal SYBR qPCR Master Mix (Vazyme, R511-03) with the primers listed in Table 3, where primers M-Actb-F and M-Actb-R were used to amplify the internal control gene β-actin.

### Determination of CXCL-1, CXCL-2, CCL-3, CCL-4, and CCL-5 in the peritoneal lavage and serum collected from mice treated with cathelicidins

A total of 45 C57BL/6J mice (6–8 weeks, female) were randomly assigned into 9 groups with 5 mice per group and were intraperitoneally administered cCATH-1, cCATH-3, or PBS (200 μL) at a dose of 10 mg/kg body weight. Orbital sinus blood and peritoneal lavage fluid were collected at 4, 8, 12, and 24 h after inoculation, and serum and peritoneal lavage supernatants were obtained following centrifugation. The protein levels of CXCL-1, CXCL-2, CCL-3, CCL-4, and CCL-5 were measured using the commercial ELISA kits described above [16].

### Determination of the chemotactic effect of the recombinant mouse CCL-3(mCCL3) protein on mouse peritoneal macrophages and the neutralizing ability of the anti-mCCL3 polyclonal antibody

#### Expression of the recombinant mCCL3

Using primers mCCL3-F (ATACCATGGCGCCGTATGGAGCTGACACCCCGACTGCCTGCTGCTTCTCCTACAGCCGT) and mCCL3-R (GTGCTCGAGTCAGGCATTCAGTTCCAGGTCAGTGATGTATTCTTGGACCCAGGTCTCTT), the codon-optimized *CCL3* encoding gene was cloned into pET28a for expression and subsequently expressed and purified as described in previous studies [40, 41].

#### Determination of chemotactic effect of the mCCL3 protein on mouse peritoneal macrophages

In a 24-well Transwell cell culture system (5 μm pore size), 5 × 10^5^ mouse peritoneal macrophages/well (5 × 10^6^ cells/mL, 0.1 mL) were seeded in the upper chamber, and 500 μL of RPMI 1640 containing 4% FBS was added to the lower chamber. mCCL3 protein was added to the lower chamber at concentrations of 0, 0.1, 0.2, 0.5, 1, 2, 5, 10, 20, 50, and 100 ng/mL, and incubated at 37 °C with 5% CO_2_ for 8 h. The number of cells that migrated to the lower chamber was counted, and the ED_50_ (50% effective dose) of mCCL3 protein was calculated.

#### Development of rabbit anti-mCCL3 polyclonal antibody

Rabbits were administered subcutaneously with mCCL3 protein or vector control pET28a/BL21(DE3) protein (initial immunogen emulsified with Freund’s complete adjuvant), 500 μg protein/rabbit at the first immunization, and 15 days later, subsequent boost immunizations were performed using immunogen emulsified with Freund’s incomplete adjuvant, 100 μg protein/rabbit at 10-day intervals for three times. Blood was collected, and the antibody titer against mCCL3 was detected by ELISA and agarose diffusion assay, and the polyclonal antibody was purified using the caprylic acid-ammonium sulfate (CAAS) precipitation method [42]. The concentration of purified IgG was determined using a BCA protein assay kit (Beyotime, Shanghai, China, P0010). Three purified serum IgGs were obtained, namely mCCL3-specific antibody IgG (referred to as mCCL3 Ab), vector control pET28a/BL21(DE3) protein-specific antibody IgG (referred to as Vec Ab), and serum IgG of mock rabbits (referred to as Mock Ab), respectively.

In vitro neutralizing activity of mCCL3 Ab was determined. A 24-well Transwell cell system (5 μm pore size) was employed, and 0.1 mL of 5 × 10^5^ mouse peritoneal macrophages were added into the upper chamber, while 500 μL of RPMI 1640 supplemented with 4% FBS was placed in the lower chamber. The mCCL3 Ab (5 mg/mL) was diluted at ratios of 1:10, 1:100, 1:1000, and 1:10,000, and then mixed with mCCL3 protein (5 μg/well) in the lower chamber. The positive control wells contained only mCCL3 protein (5 μg/well) without mCCL3 Ab, whereas the negative control wells contained neither antibody nor mCCL3 protein. The system was incubated at 37 °C with 5% CO_2_ for 8 h, and the number of cells migrating to the lower chamber was counted.

#### Characterization of mCCL3 Ab in blocking the chemotactic effect of the mCCL3 on mouse peritoneal macrophages

In vivo, the neutralizing ability of mCCL3 Ab was evaluated. mCCL3 Ab, Vec Ab, and Mock Ab were injected intraperitoneally into mice at a dose of 100 μg/mouse (200 μL). One hour later, peritoneal macrophages were collected from the mice. Cell crawling slides were placed in the cell culture plate, and cells were allowed to adhere for 2 h before immunofluorescence assay (IFA). The cells on slides were fixed with 4% pre-cooled paraformaldehyde at room temperature for 20 min. For positive control, fixed untreated cells were incubated with 5 μg mCCL3 protein for 1 h. Fixed peritoneal macrophages from mice treated with mCCL3 Ab, Vec Ab, Mock Ab, or positive control were incubated with 200 μL of mCCL3 Ab (0.5 mg/mL) at 37 °C for 1 h. After washing three times with PBS, FITC-labeled goat anti-rabbit IgG (H+L) (1:500 dilution, Beyotime, A0562) was added and incubated at 37 °C for 1 h. After washing three times with PBS, a far-red fluorescent dye cell membrane staining kit (DiD) (1:200 dilution, Beyotime, C1995S) was used for incubation at 37 °C for 1 h to label the cell membrane with red fluorescence. Before observation, 5 μL of antifade mounting medium with DAPI (Beyotime, P0131) was added. Samples were observed under an LSM 880NLO two-photon laser confocal microscope (Carl Zeiss AG, Baden-Württemberg, Federal Republic of Germany), and semi-quantitative analysis was performed using ZEN 3.4 software [43].

#### *Efficacy of mCCL3 Ab in cathelicidin*-*treated mice challenged with APEC E058 strain*

C57BL/6J mice (6–8 weeks old, female) were randomly divided into 11 groups with five mice per group (Table 4). Mice in groups 1, 4, and 7 were intraperitoneally administered cCATH-1 at 10 mg/kg (200 μL) 4 h before APEC challenge. Mice in groups 2, 5, and 8 were intraperitoneally administered cCATH-3 at 10 mg/kg body weight (200 μL) 4 h prior to challenge. Mice in groups 1–3 were administered mCCL3 Ab (100 μg/mouse) 1 h before challenge, followed by an additional administration of 150 μg/mouse of the same antibody at 24 h post-challenge. Mice in groups 4–6 were intraperitoneally injected with Vec Ab (100 μg/mouse), and subsequent treatment with Vec Ab was the same as mCCL3 Ab in groups 1–3. Mice in groups 3, 6, and 9 were intraperitoneally injected with 200 μL of PBS 4 h before challenge. Mice in group 10 were intraperitoneally injected with mCCL3 protein (5 μg/mouse) (200 μL) 4 h prior to challenge. Mice in group 11 were left untreated as a healthy control. Mice in groups 1–10 were challenged intraperitoneally with 4 × 10^6^ CFU/mouse of APEC E058 strain (200 μL), while mice in group 11 were inoculated intraperitoneally with 200 μL of PBS. All mice were observed, and mortality was recorded continuously for one week following challenge [44].

**Table 4:** **Groups and treatments to evaluate the effect of mCCL3 Ab on protection of cathelicidins in mice against APEC E058 strain challenge**

Groups	4 h before challenge	1 h before challenge	24 h after challenge	APEC E058 strain challenge
1	10 mg/kg cCATH-1 (200 μL)	mCCL3 Ab (100 μg/mouse)	mCCL3 Ab (150 μg/mouse)	+
2	10 mg/kg cCATH-3 (200 μL)	mCCL3 Ab (100 μg/mouse)	mCCL3 Ab (150 μg/mouse)	+
3	PBS (200 μL)	mCCL3 Ab (100 μg/mouse)	mCCL3 Ab (150 μg/mouse)	+
4	10 mg/kg cCATH-1 (200 μL)	Vec Ab (100 μg/mouse)	Vec Ab (150 μg/mouse)	+
5	10 mg/kg cCATH-3 (200 μL)	Vec Ab (100 μg/mouse)	Vec Ab (150 μg/mouse)	+
6	PBS (200 μL)	Vec Ab (100 μg/mouse)	Vec Ab (150 μg/mouse)	+
7	10 mg/kg cCATH-1 (200 μL)	–		+
8	10 mg/kg cCATH-3 (200 μL)	–		+
9	PBS (200 μL)	–		+
10	mCCL3 protein (5 μg/mouse) (200 μL)	–		+
11	–	–		–

“+” means involved, and “–” means not involved.

### Statistical analysis

Statistical analysis was performed using GraphPad Prism 8 (GraphPad Software, USA) and IBM SPSS (Statistical Package for the Social Sciences) Statistics 22. Significant differences (*p* < 0.05) among the means of three or more groups were analyzed using one-way analysis of variance (ANOVA) followed by the least significant difference (LSD) multiple-comparison test. Fisher’s exact tests were used to compare mortality rates among different groups. A *P*-value of less than 0.05 was considered statistically significant.

## Results

### Antimicrobial peptide synthesis

**Figure 1:**
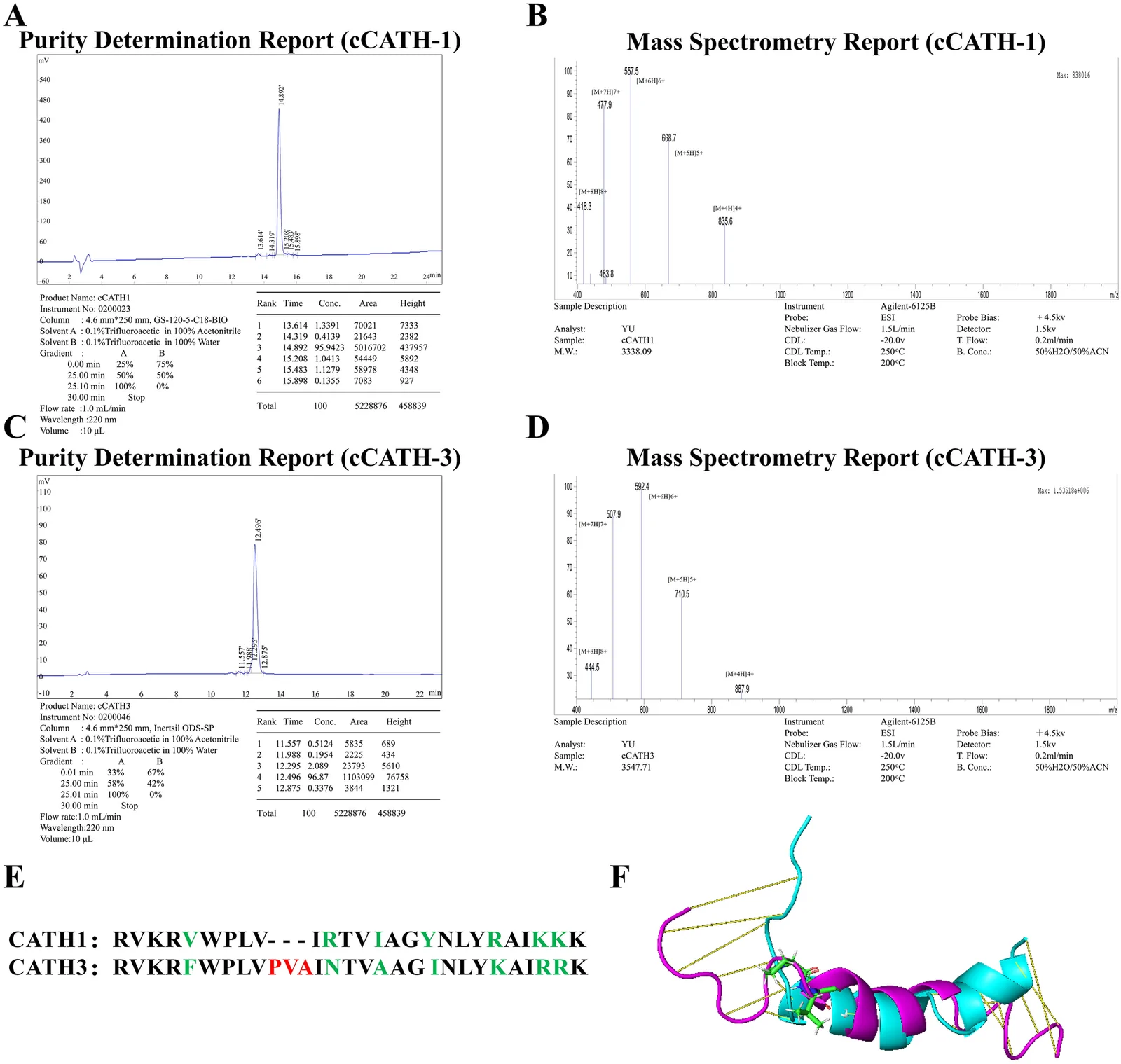
**Purity and molecular weight of cathelicidins.** **A** Purity of cCATH-1; **B** Mass spectrometry of cCATH-1; **C** Purity of cCATH-3; **D** Mass spectrometry of cCATH-3; **E** Alignment between cCATH-1 and cCATH-3, amino acids colored red indicate gaps, green indicate differences, and black indicate identity; **F** The cCATH-1 structure (PDB ID: 2AMN [45]) with two adjacent α-helices is shown in blue, and the cCATH-3 structure (PDB ID:2HFR [46]) with one α-helix is shown in purple.

The purity of cCATH-1 was 96.89% (Figure 1A), and its molecular weight was 3338.09 Da (Figure 1B). The purity of cCATH-3 was 96.82% (Figure 1C), and its molecular weight was 3547.71 Da (Figure 1D).

The amino acid alignment between cCATH-1 and cCATH-3 (Figure 1E) showed a high sequence identity of 60% between these two cathelicidin orthologs. Although cCATH-3 had three more amino acids than cCATH-1, their overall structures were similar (Figure 1F).

### Determination of the minimum inhibitory concentration (MIC) of synthesized cCATH-1 and cCATH-3

The MIC results indicated that cCATH-1 was 4- to 16-times less than those of cCATH-3 against APEC isolates (Table 5). This difference likely reflects their differential susceptibility to APEC proteases: our unpublished data show that E058 cOmpT/pOmpT cleave cCATH-3 but not cCATH-1, reducing cCATH-3’s effective concentration in vitro. Notably, this protease-mediated inactivation does not diminish cCATH-3’s in vivo efficacy, implying that in vitro MIC alone is not necessary to predict its in vivo protection in this case.

**Table 5:** **The MIC of cCATH-1 and cCATH-3 against different bacteria**

	cCATH-1 – μM (μg/mL)^*^	cCATH-3 – μM (μg/mL)^*^
pET28a/BL21	2.5 (8.36)	2.5 (8.87)
pET28a-c*ompT*/BL21(DE3)	2.5 (8.36)	10 (35.5)
pET28a-p*ompT*/BL21(DE3)	2.5 (8.36)	5 (17.75)
ATCC25922	2.5 (8.36)	5 (17.75)
*E. coli* E516 (serogroup *O1*)	5 (16.72)	20 (70.94)
*E. coli* E058 (serogroup *O2*)	5 (16.72)	80 (283.78)
*E. coli* E522 (serogroup *O78*)	10 (33.43)	40 (141.89)

^*^The concentration conversion was based on the mass spectrometry data.

### HC_50_ of synthesized cCATH-1 and cCATH-3

The cytotoxicity of cCATH-1 and cCATH-3 on chicken erythrocytes was determined. The HC_50_ values of cCATH-1 and cCATH-3 were 834.776 μg/mL (249 μM) and 2873.885 μg/mL (810 μM), respectively (Table 6). cCATH-3 is less hemolytic and relatively safer than cCATH-1 toward chicken erythrocytes. The concentrations used in this study for cells, mice, etc., were all much lower than their semi-hemolytic concentrations and can be considered safe.

**Table 6:** **The HC of**_50_ **cCATH-1 and cCATH-3 against chicken erythrocytes**

	HC_50_ – μM (μg/mL)
cCATH-1	249 (834.776)
cCATH-3	810 (2873.885)

### Ultrastructure observation of APEC E058 strain treated with cathelicidins

**Figure 2:**
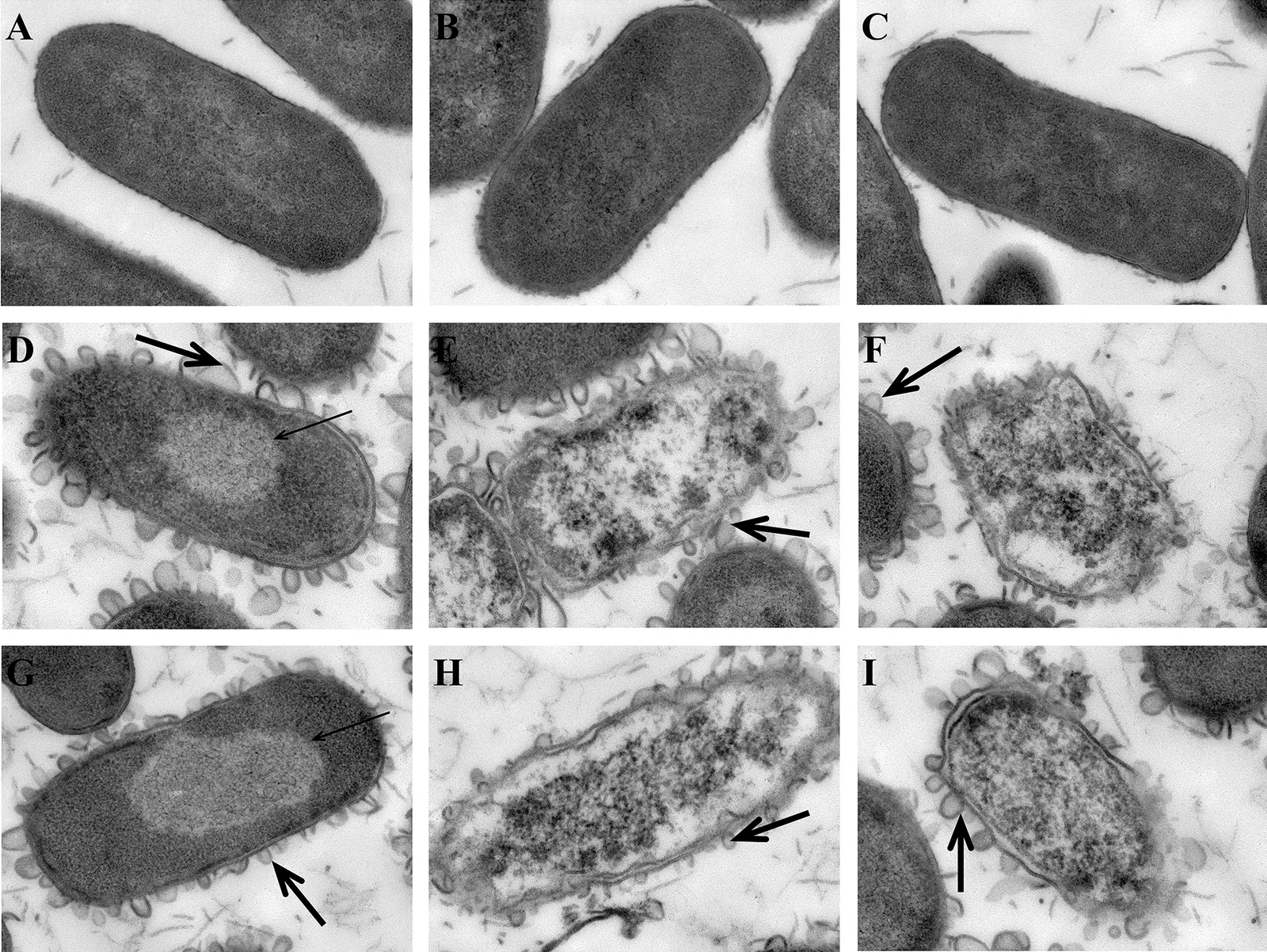
**Ultrathin sections of bacteria treated with cathelicidins.** **A**–**C** Untreated APEC E058 strain; **D**–**F** cCATH-1-treated E058 strain for 1 h; **G**–**I** cCATH-3-treated E058 strain for 1 h. Bold arrows indicate vesicles, and thin arrows indicate aggregation of nuclei.

Untreated APEC E058 strain showed intact morphology (Figure 2A, B, and C). After treatment with either cCATH-1 or cCATH-3, vesicles were formed on the bacterial surface (bold arrows), suggesting membrane leakage, while aggregation of nuclei was also observed (thin arrows) (Figure 2D, G). Interestingly, partial bacteria were severely damaged, resulting in almost complete loss of intracellular contents, and eventually the bacteria were disrupted and killed (Figure 2E, F, H, I). Therefore, it can be concluded that the killing effect of cCATH-1 or cCATH-3 on APEC E058 strain in vitro is mainly achieved by disrupting the cell membrane and causing lesions in the nucleus and cytoplasm.

### Protection of cCATH-1/cCATH-3 against APEC E058 strain challenge in mice

**Figure 3:**
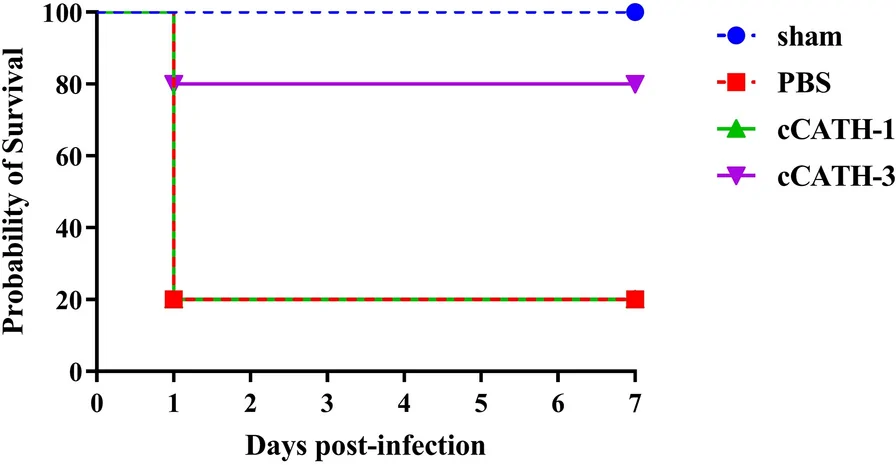
**Survival rate of mice post-challenge.** Female C57BL/6 J mice (6–8 weeks old, n = 5 per group) were randomly assigned to four groups: sham (PBS only), PBS challenge control (PBS pretreatment + APEC E058), cCATH-1 (10 mg/kg + APEC E058), and cCATH-3 (10 mg/kg + APEC E058). Inoculates of pretreatments were administered intraperitoneally 4 h prior to bacterial challenge (4 × 10^6^ CFU/mouse). Mortality was monitored for 7 days post-challenge.

There was no death of mice in the mock group. The survival rate of mice in the challenge-only group and in the cCATH-1-treated group was only 20%; however, the survival rate of mice in the cCATH-3-treated group reached 80% (Table 7, Figure 3). These results indicated that cCATH-1, despite strong bacteriocidal ability in vitro, did not show effective protection in vivo, whereas cCATH-3, with relatively weaker bacteriocidal activity in vitro, achieved markedly higher protection compared with cCATH-1, implying that cCATH-3 employs additional mechanisms beyond direct bacteriocidal activity to protect mice against APEC challenge.

**Table 7:** **Survival rate of mice post-challenge**

	Days post-challenge						
	1	2	3	4	5	6	7
cCATH-1	1/5	1/5	1/5	1/5	1/5	1/5	1/5
cCATH-3	5/5	4/5	4/5	4/5	4/5	4/5	4/5
Challenge control	1/5	1/5	1/5	1/5	1/5	1/5	1/5
Sham	5/5	5/5	5/5	5/5	5/5	5/5	5/5

### Differential cell populations of mice treated with cathelicidins

**Figure 4:**
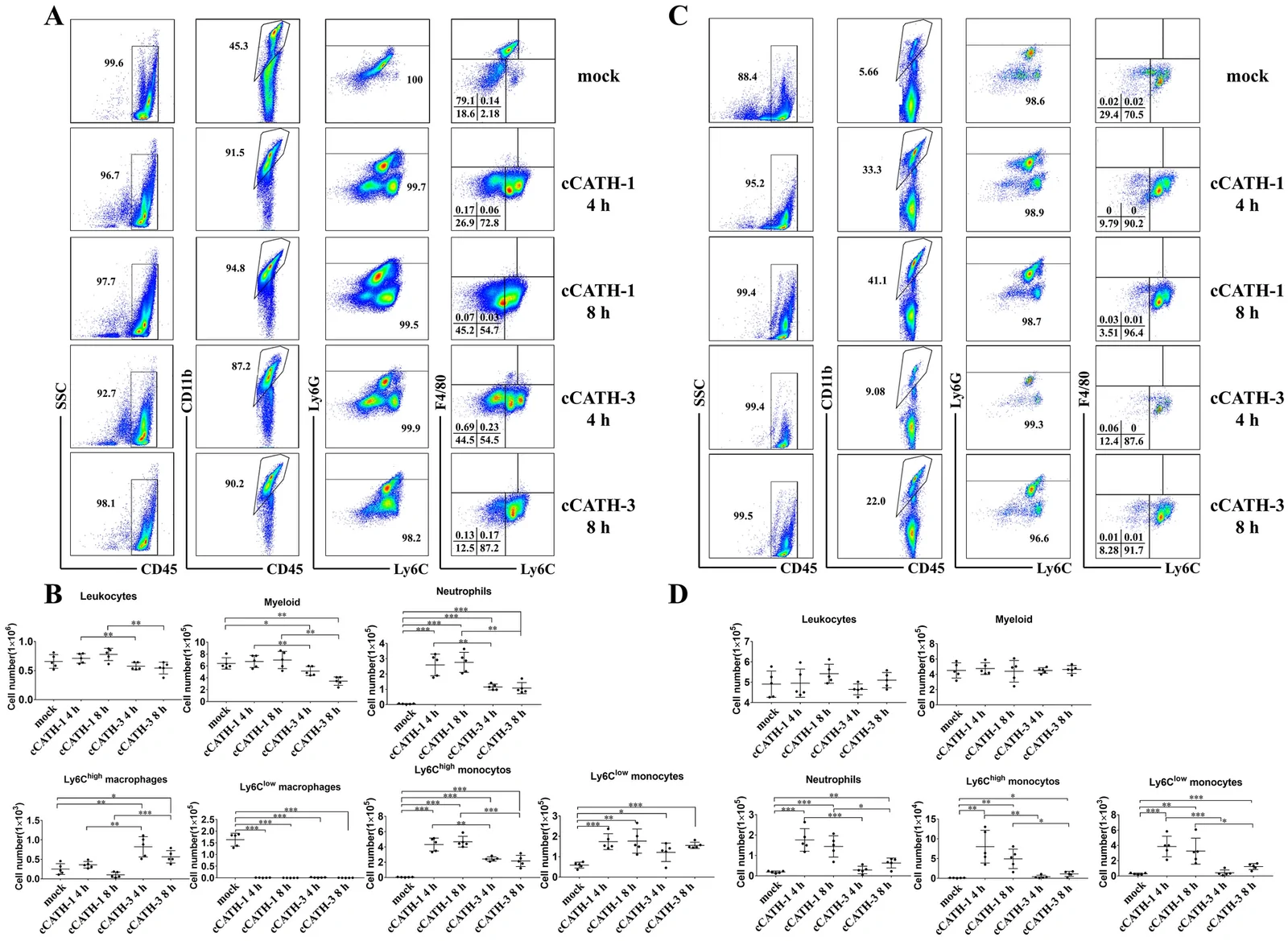
**The proportion of cells sorted by flow cytometry as well as the number of various cell types.** C57BL/6 J female mice (6–8 weeks old, n = 5 per group) were intraperitoneally injected with cCATH-1 (10 mg/kg), cCATH-3 (10 mg/kg), or PBS. Peritoneal lavage fluid and peripheral blood were collected at 4 and 8 h post-treatment. **A** Proportions of myeloid cells in peritoneal lavage from mice treated with or without cCATH-1 or cCATH-3 for 4 and 8 h; **B** Proportion of different cells in peripheral blood from mice treated with or without cCATH-1 or cCATH-3 for 4 and 8 h; **C** Numbers (per million cells) of myeloid cells in peritoneal lavage from mice treated with or without cCATH-1 or cCATH-3 for 4 and 8 h; **D** Numbers (per million cells) of myeloid cells in peripheral blood from mice treated with or without cCATH-1 or cCATH-3 for 4 and 8 h.

The total counts of white blood cells, myeloid cells, and neutrophils in both peritoneal lavage fluid and peripheral blood from cCATH-3-treated mice were significantly lower than those in cCATH-1-treated mice at 4 and 8 h after treatment (*p* < 0.01) (Figure 4A, B). For peritoneal lavage fluid, no significant difference was observed in the total numbers of white blood cells and myeloid cells between the cCATH-1-treated group and the mock notably, the total number of myeloid cells in the cCATH-3-treated group was markedly lower than that in the other two groups (*p* < 0.01) (Figure 4A, B). Although the number of neutrophils in the cCATH-3-treated group was significantly higher than that in the mock group (*p* < 0.001) (Figure 4A, B), it remained clearly lower than that in the cCATH-1-treated group (*p* < 0.01) (Figure 4A, B). In peripheral blood, no significant difference was found in the total numbers of white blood cells and myeloid cells among cCATH-1, cCATH-3, and mock groups, although the total number of neutrophils in the cCATH-1- and cCATH-3-treated groups was significantly higher than that in the mock group (*p* < 0.05), except for the group at 4 h after cCATH-3 treatment (Figure 4C, D). However, the total number of neutrophils remained significantly lower in the cCATH-3-treated group compared with that in the cCATH-1-treated group (*p* < 0.05 or *p* < 0.001) (Figure 4C, D).

Meanwhile, in the peritoneal lavage fluid of mice, the Ly6C^high^ macrophage subset in the cCATH-3-treated group was markedly higher than that in the cCATH-1-treated group at least at 4 and 8 h after treatment (*p* < 0.01 or *p* < 0.001) (Figure 4A, B), whereas no significant difference was observed in the Ly6C^low^ macrophage subset between the two groups at the examined time points.

Although the total number of myeloid cells decreased in cCATH-3-treated mice compared with that in cCATH-1-treated mice, the number of Ly6C^high^ macrophage subsets in these mice significantly increased relative to cCATH-1-treated mice, implying that Ly6C^high^ macrophages play an unclear role in the protection of cCATH-3 in mice against APEC infection.

### Phagocytosis of APEC E058 strain by mouse peritoneal macrophages treated with cCATH-1 or cCATH-3

**Figure 5:**
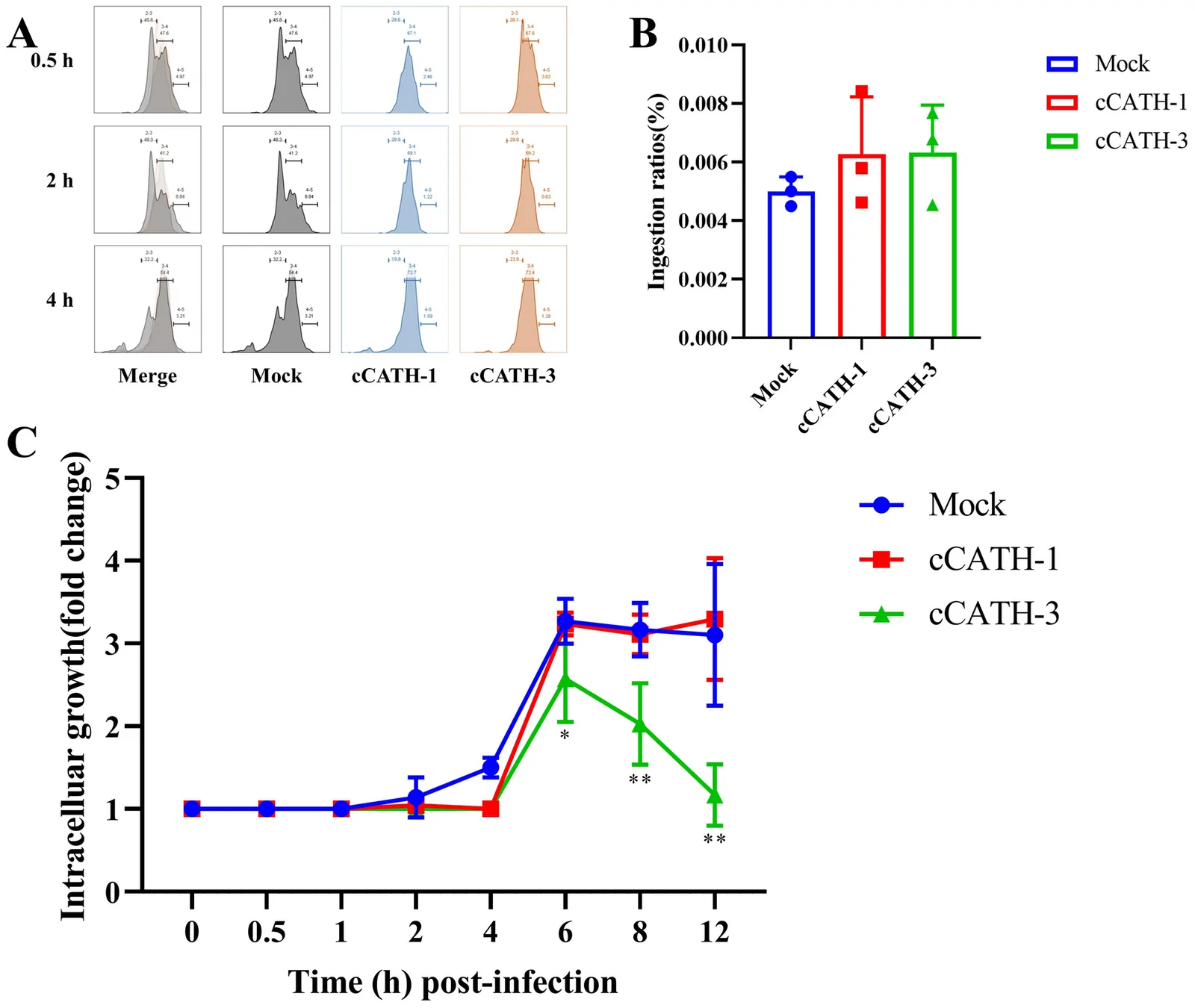
**Phagocytosis and intracellular killing of APEC E058 by mouse peritoneal macrophages treated with cCATH-1 or cCATH-3.** **A** Representative flow cytometry histograms showing CFSE fluorescence in macrophages incubated with inactivated, CFSE-stained APEC E058 for 0.5, 2, and 4 h. **B** Quantification of phagocytic efficiency determined by plate counting. **C** Intracellular bacterial survival over time (0–12 h) following phagocytosis of live APEC E058. Data in **B** and **C** represent mean ± SD from three independent experiments (*n* = 3 biological replicates per group). Statistical significance in (**B**) was determined by one-way ANOVA with LSD post-hoc test; in (**C**), two-way ANOVA with Bonferroni post-hoc test was used to compare cCATH-3-treated versus cCATH-1-treated groups at each time point. * *p* < 0.05 and ** *p* < 0.01 indicate significance between cCATH-3-treated and cCATH-1-treated peritoneal macrophages.

The phagocytic capacity of peritoneal macrophages pretreated with cCATH-1 or cCATH-3 for 2 h was significantly higher than that of untreated cells during 4 h incubation with fluorescence-stained inactivated bacteria (*p* < 0.05) (Figure 5A). However, there was no significant difference in the amount of inactivated bacteria phagocytosed by peritoneal macrophages treated with either cCATH-1 or cCATH-3 (Figure 5A).

The phagocytic ability toward live APEC E058 bacteria in peritoneal macrophages treated with cathelicidins was not statistically significant compared with that of untreated cells (Figure 5B).

The live APEC E058 strain loads in peritoneal macrophages treated with cCATH-1 were comparable to those in untreated macrophages; however, the live bacteria in cells treated with cCATH-3 significantly decreased at 6–12 h post-infection compared with cCATH-1-treated or untreated macrophages (*p* < 0.05 or *p* < 0.01), indicating that cCATH-3-treated peritoneal macrophages eliminated more E058 cells compared with cCATH-1-treated cells in vitro (Figure 5C).

### Chemotactic effect of cCATH-3 on mouse peritoneal macrophages

**Figure 6:**
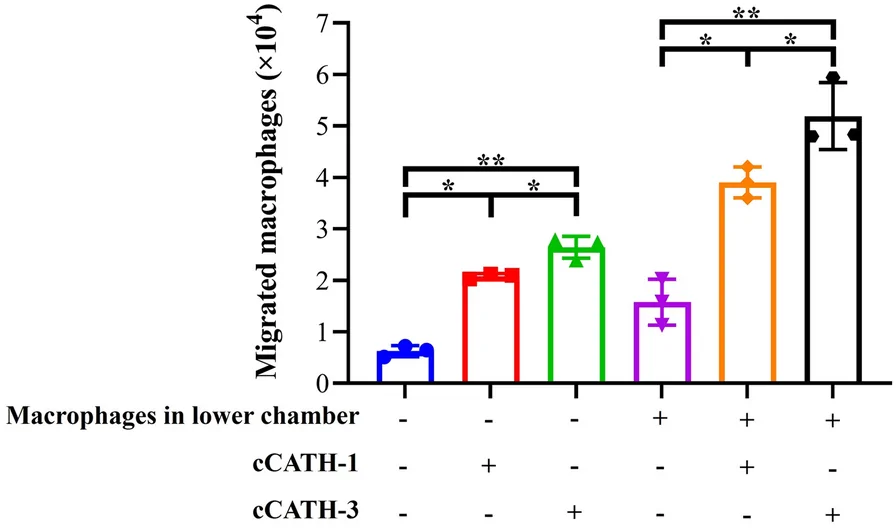
**In vitro chemotaxis effect of cCATH-1 or cCATH-3 on mouse peritoneal macrophages.** Macrophages (5 × 10⁶ cells/mL, 100 μL) were seeded in the upper chamber of a 5.0 μm Transwell insert, with 500 μL RPMI 1640/4% FBS in the lower chamber. In the latter, the inoculates including: no peptide or macrophages; cCATH-1 or cCATH-3 only; pre-seeded macrophages only; or pre-seeded macrophages plus cCATH-1 or cCATH-3. After 8 h at 37 °C, 5% CO₂, migrated cells were counted. Data are mean ± SD (*n* = 3). *p < 0.05, **p < 0.01.

In the single chemotaxis assay, the chemotactic effect of both cCATH-1 and cCATH-3 on peritoneal macrophages was significantly increased compared with that of mock cells (*p* < 0.05 or *p* < 0.01), and cCATH-3 exhibited stronger chemotactic capacity than cCATH-1 (*p* < 0.05) (Figure 6).

In the co-chemotaxis assay, the chemotactic capacity of both cCATH-1 and cCATH-3 groups was also significantly increased compared with that of mock cells (*p* < 0.05 or *p* < 0.01), and cCATH-3 showed stronger chemotactic activity than cCATH-1 (*p* < 0.05) (Figure 6).

These results indicated that both cCATH-1 and cCATH-3 were able to recruit mouse peritoneal macrophages, and cCATH-3 exhibited stronger chemotactic ability than cCATH-1.

### Macrophages are essential for the shelter of cCATH-3 in mice against APEC challenge

**Figure 7:**
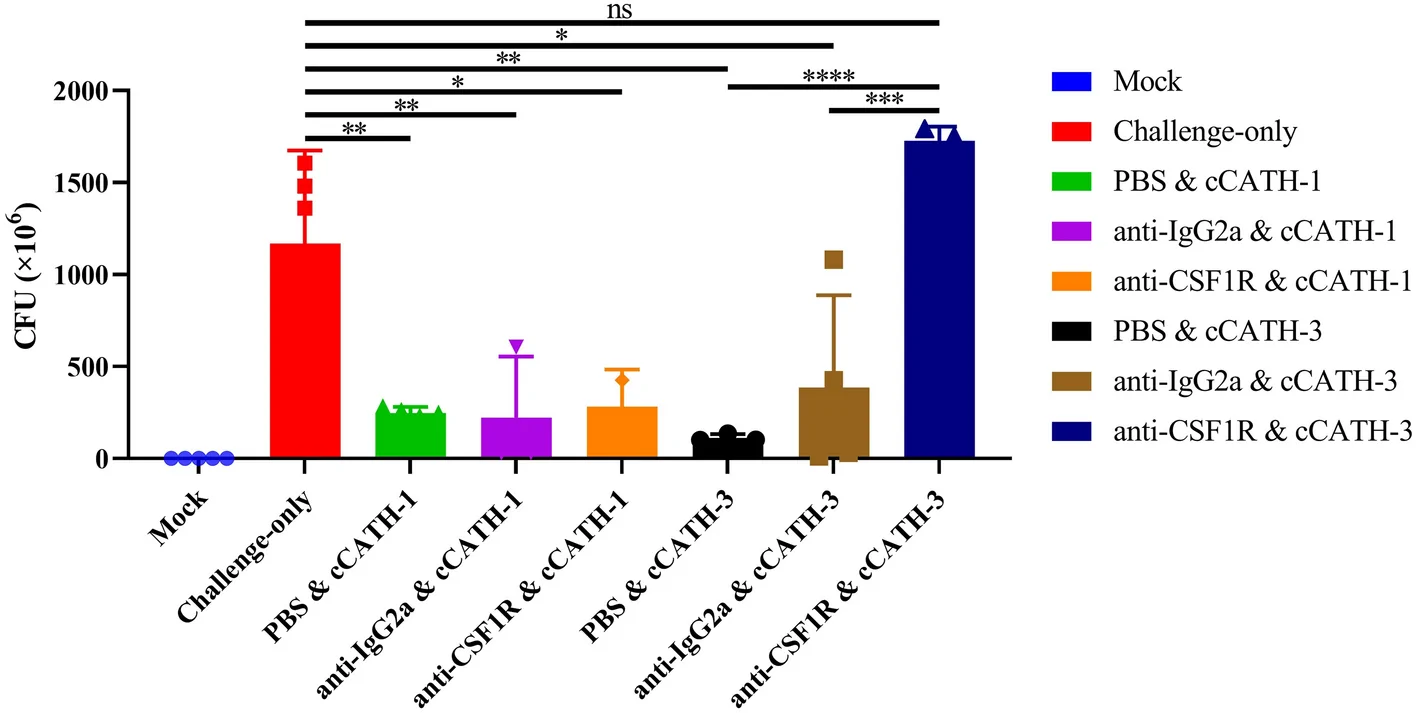
**Intraperitoneal bacterial load in mice treated with different antibodies at 18 h post-challenge.** C57BL/6 J mice (n = 5 per group) were intraperitoneally administered anti-CSF1R antibody (1 mg on day 0, 0.3 mg on day 1, day 2, respectively), IgG2a isotype control, or PBS. On day 3, cCATH-1 or cCATH-3 (10 mg/kg) was intraperitoneally injected 4 h before bacterial challenge (4 × 10⁶ CFU). Peritoneal lavage fluid was collected at 18 h post-challenge for CFU enumeration. Data are mean ± SD (n = 5). ns, not significant; *p < 0.05, **p < 0.01, ***p < 0.001.

There was no significant difference in bacterial load in cCATH-1-treated mice regardless of the type of antibody employed, implying that cCATH-1 might have the ability to eliminate intraperitoneal bacteria independent of macrophages (Figure 7). Bacterial loads in PBS & cCATH-1-treated mice and PBS & cCATH-3-treated mice remained comparable, and were significantly lower than those in challenge-only mice after APEC E058 challenge (*p* < 0.01) (Figure 7). Notably, there was no significant difference between challenge-only mice and anti-CSF1R & cCATH-3-treated mice (Figure 7), given that macrophages were depleted in anti-CSF1R & cCATH-3-treated mice, suggesting that the ability of cCATH-3 to reduce the bacterial load of APEC E058 in the peritoneal cavity was markedly abolished due to macrophage depletion [18]. These findings further indicate that macrophages targeted by anti-CSF1R antibody may represent key effector cells of cCATH-3 in mice during APEC infection.

### Cytokines produced by murine peritoneal macrophages treated with cathelicidins and/or challenged with APEC E058 strain

**Figure 8:**
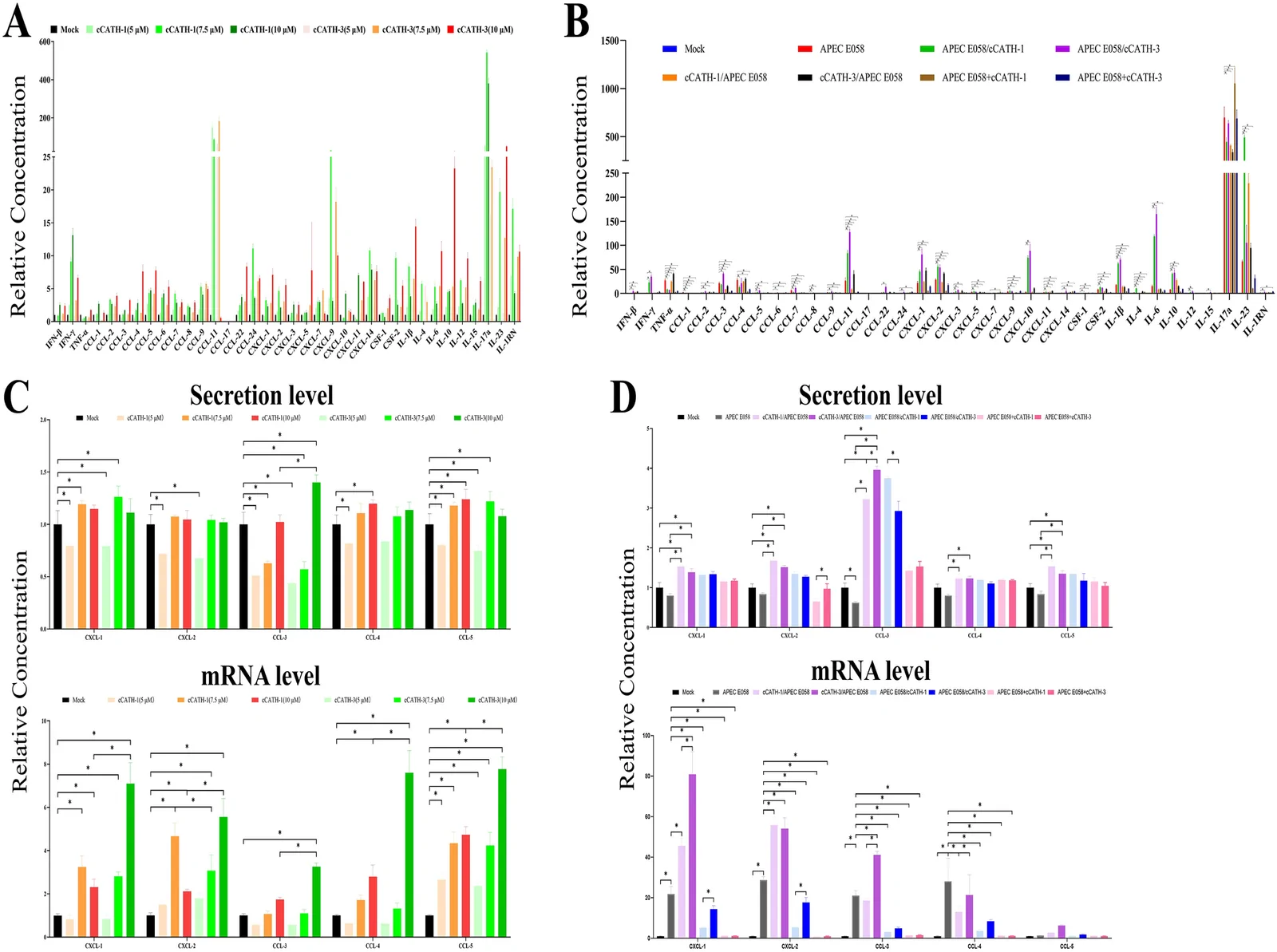
**The changes in cytokines of murine peritoneal macrophages/supernatants treated with different cathelicidins at the secretion and mRNA levels.** **A** Transcriptional levels of cytokines in mouse peritoneal macrophages treated with cCATH-1 or cCATH-3 at 5, 7.5, or 10 μM for 4 h determined by qPCR. **B** Transcriptional levels of cytokines in mouse peritoneal macrophages challenged with APEC E058 strain with or without cCATH-1/cCATH-3 treatment determined by qPCR. **C** Both transcriptional and secretion levels of cytokines CXCL-1, CXCL-2, CCL-3, CCL-4, and CCL-5 in mouse peritoneal macrophages treated with cCATH-1/cCATH-3 at different concentrations. The top panel shows the secretion levels of five cytokines in culture supernatants determined using commercial ELISA kits, and the bottom panel shows the transcriptional levels of these cytokines determined by qPCR. *(*p* < 0.05) represents significant differences between mouse peritoneal macrophages treated with different doses of cathelicidins and mock cells, or between cCATH-1-treated and cCATH-3-treated cells. **D** Both transcriptional and secretion levels of cytokines CXCL-1, CXCL-2, CCL-3, CCL-4, and CCL-5 in mouse peritoneal macrophages challenged with APEC E058 strain and pre-treated or post-treated with cCATH-1/cCATH-3. The top panel shows the secretion levels of cytokines in culture supernatants determined by commercial ELISA kits, and the bottom panel shows the transcriptional levels determined by qPCR. *(*p* < 0.05) represents significant differences between cells treated with cathelicidins, challenged with APEC E058 strain and mock cells, or between cCATH-1-treated/APEC-challenged cells and cCATH-3-treated/APEC-challenged cells.

Various chemokines and other cytokines at the transcriptional level were determined by qPCR in mouse peritoneal macrophages treated with cCATH-1 or cCATH-3 alone, pre-treated with cathelicidins followed by APEC E058 strain challenge, challenged with APEC E058 strain followed by cathelicidin treatment, and challenged with APEC E058 strain alone, respectively (Figure 8).

The transcriptional levels of cytokines in mouse peritoneal macrophages treated with cCATH-1 or cCATH-3 at 5, 7.5, or 10 μM for 4 h were determined by qPCR. Most tested chemokines and cytokines were upregulated in peritoneal macrophages treated with either cathelicidin compared with mock cells, and this increase was dose-dependent (Figure 8A). Furthermore, chemokines and cytokines, including IL-1β, IL-12, IL-10, IL-17a, IL-23, IFN-γ, CXCL-1, CXCL-2, CCL-3, CCL-4, and CCL-5, were upregulated in peritoneal macrophages treated with cathelicidins and subjected to APEC challenge compared with APEC challenge-only cells, regardless of whether cathelicidin treatment preceded or followed APEC challenge (Figure 8B) (*p* < 0.05).

The secretion levels of cytokines CXCL-1, CXCL-2, CCL-3, CCL-4, and CCL-5 in mouse peritoneal macrophages treated with cCATH-1/cCATH-3 at different concentrations were determined using commercial ELISA kits. The CCL-3 secretion level in cells treated with 10 μM cCATH-3 was significantly higher than that in cells treated with cCATH-1 at the same concentration (*p* < 0.05), consistent with the transcriptional level (*p* < 0.05) (Figure 8C). However, the levels of CXCL-1, CXCL-2, CCL-4, and CCL-5 in mouse peritoneal macrophages treated with cCATH-1/cCATH-3 varied with both the type and concentration of cathelicidins, without a clear trend between cCATH-1-treated and cCATH-3-treated cells (Figure 8C).

To examine the levels of CXCL-1, CXCL-2, CCL-3, CCL-4, and CCL-5 in mouse peritoneal macrophages challenged with APEC E058 strain before or after cCATH-1/cCATH-3 treatment, a cell model combining cathelicidin treatment and APEC challenge was used. When mouse peritoneal macrophages were treated with cCATH-3 for 4 h prior to APEC E058 challenge, the CCL-3 secretion level was significantly higher than that in cCATH-1-treated cells (*p* < 0.05), consistent with the CCL-3 transcriptional level in the 10 μM cCATH-1-treated group (*p* < 0.05). However, the CCL-3 secretion level in APEC-challenged cells subsequently treated with cCATH-1 was significantly higher than that in cCATH-3-treated cells (*p* < 0.05) (Figure 8D). Meanwhile, the levels of CXCL-1, CXCL-2, CCL-4, and CCL-5 in mouse peritoneal macrophages pre-treated with cCATH-3 for 4 h before APEC challenge were comparable to those in cCATH-1-treated cells (Figure 8D). These results suggest that cytokine CCL-3 induced by cCATH-3 may play a central role in the host response against APEC infection.

### cCATH-3 induces higher CCL-3 and CCL-5 levels than cCATH-1 in mouse peritoneal lavage and serum

**Figure 9:**
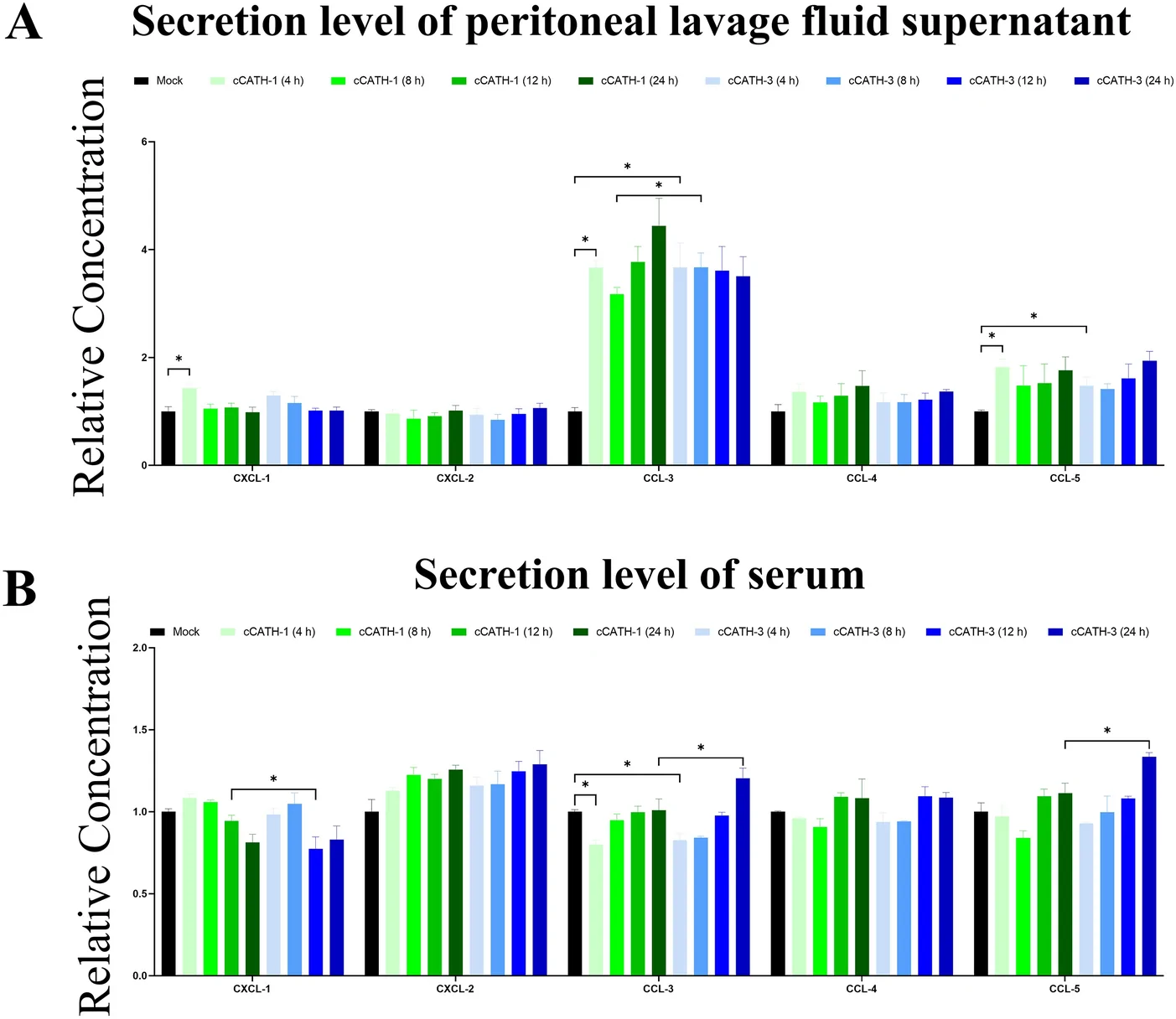
**Secretion level of chemokines CXCL-1, CXCL-2, CCL-3, CCL-4, and CCL-5 in peritoneal lavage and serum from mice treated with different doses of cCATH-1/cCATH-3.** **A** Secretion levels of chemokines CXCL-1, CXCL-2, CCL-3, CCL-4, and CCL-5 in peritoneal lavage determined by commercial ELISA kits. **B** Secretion levels of these chemokines in serum determined by commercial ELISA kits. *(*p* < 0.05) represents significant differences between mice treated with different doses of cathelicidins and mock mice, or between cCATH-1-treated and cCATH-3-treated mice.

The levels of CXCL-1, CXCL-2, CCL-3, CCL-4, and CCL-5 chemokines in both the peritoneal lavage and the serum collected from mice treated with cathelicidins were determined by ELISA assay. Differences in the levels of these five chemokines in the supernatant of peritoneal lavage fluid collected from mice treated with different doses of cathelicidins were observed (Figure 9A).

In the supernatant of peritoneal lavage fluid, CXCL-1 showed a transient increase at 4 h after cCATH-1 treatment, while CXCL-2 and CCL-4 remained relatively stable. However, the secretion levels of CCL-3 and CCL-5 in the peritoneal lavage fluid of mice treated with either cathelicidin for 4 h were significantly higher than those of mock mice (*p* < 0.05), and CCL-3 in the cCATH-3 group was significantly higher than that in the cCATH-1 group at 8 h after treatment (Figure 9A). In serum, the levels of CCL-3 and CCL-5 in mice treated with cCATH-3 were significantly higher than those in mice treated with cCATH-1 at 24 h (*p* < 0.05) (Figure 9B). Overall, among the measured chemokines in both peritoneal lavage fluid and serum, only CXCL-1, CCL-3, and CCL-5 showed significant differences between cCATH-1 and cCATH-3 treatments (Figure 9).

### Effect of mCCL3 Ab on protection of cCATH-3 in mice challenged with APEC strain

Mice were treated with cCATH-3 or mCCL3 protein 4 h before challenge, followed by treatment with or without mCCL3 Ab or Vec Ab 1 h before challenge, and the survival rate of mice was determined after challenge with APEC E058 strain.

**Figure 10:**
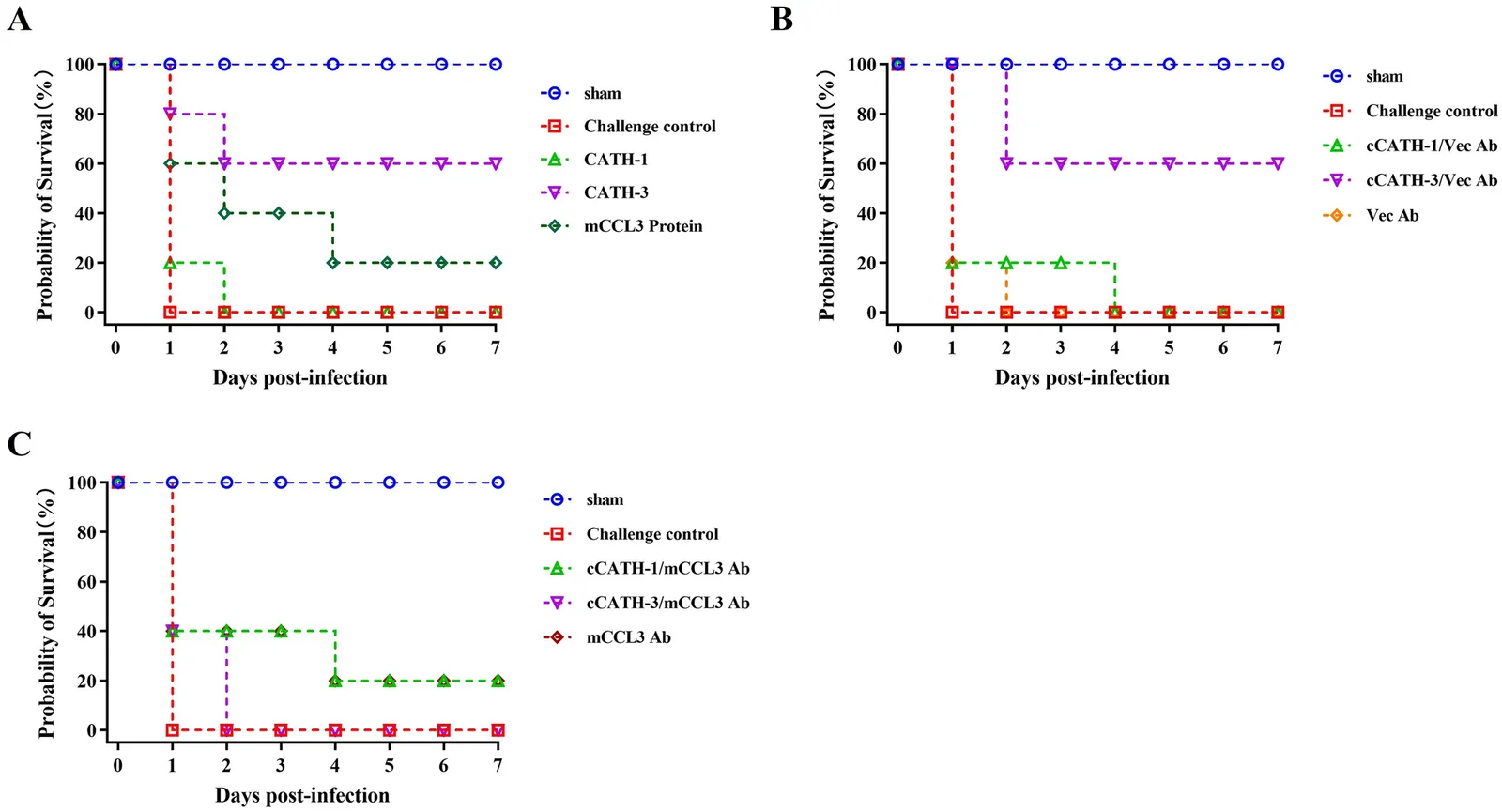
**Survival rate of mice treated with cathelicidins or mCCL3 protein, and with or without mCCL3 Ab or Vec Ab after challenge with APEC E058 strain.** **A** Survival rate of mice treated with cCATH-1, cCATH-3, and mCCL3 recombinant protein after challenge. **B** Survival rate of mice treated with both cathelicidins and Vec-Ab after challenge. **C** Survival rate of mice treated with both cathelicidins and mCCL3 Ab after challenge.

The survival rate of mice treated with cCATH-3 was 60%, whereas mice treated with cCATH-1 or subjected to challenge only died after challenge (Figure 10A). When treated with Vec Ab, the survival rate of mice treated with cCATH-3 remained 60%, and that of mice treated with cCATH-1 was 0% after challenge, consistent with the survival rates of mice without antibody treatment, suggesting that Vec Ab had no effect on the protection of cathelicidins in mice against APEC challenge (Figure 10B). Notably, the survival rate of mice treated with both cCATH-3 and mCCL3 Ab was 0%, and that of mice treated with both cCATH-1 and mCCL3 Ab was 20% (Figure 10C), indicating that the protection of cCATH-3 in mice against APEC infection was completely abolished by mCCL3 Ab neutralization. In addition, treatment with mCCL3 protein 4 h before challenge appeared to confer limited protection against APEC infection (20% survival rate versus 0% survival rate in challenge-only mice, Figure 10A).

## Discussion

This study reveals a striking gap between in vitro bacteriocidal activity and in vivo protective efficacy for two closely related avian cathelicidins. Despite sharing 60% sequence identity and similar α-helical structures [45, 46], cCATH-1 exhibited 4- to 16-fold lower MIC values than cCATH-3 against APEC isolates (Table 5), yet failed to protect mice from lethal APEC challenge, whereas cCATH-3 conferred 80% survival (Table 7, Figure 3). This gap aligns with growing evidence that direct bacteriocidal activity of HDPs is strongly influenced by physiological conditions—such as salt concentration, divalent cations, and proteolytic cleavage by bacterial virulence factors including OmpT [2, 25, 47, 48].

In cytotoxicity evaluation, the semi-hemolytic concentration (HC_50_) of cCATH-1 was lower, indicating a higher cytotoxicity compared with cCATH-3. Both peptides exerted direct bacteriocidal effects by disrupting bacterial membranes and inducing lysis, as observed by transmission electron microscopy (TEM), which further confirmed differences in membrane damage at both semi-hemolytic concentrations and cytotoxicity levels between cCATH-1 and cCATH-3 (Additional file 1).

Our findings support an emerging paradigm in HDPs biology: for certain peptides, immunomodulatory functions may outweigh direct bacteriocidal activity in vivo [12, 13, 18, 49–53]. Notably, our results are consistent with the recent report by Yang et al. [18] on PopuCATH, a non-bacteriocidal cathelicidin protects hosts by driving phagocyte influx rather than direct killing. However, unlike PopuCATH, which broadly stimulates multiple chemokines and promotes NET release, cCATH-3 appears to selectively trigger the CCL3-CCL5 axis to recruit Ly6C^high^ macrophages, suggesting a specific chemokine-mediated mechanism.

Mechanistically, cCATH-3-mediated protection potentially derived from CCL3-dependent recruitment and activation of Ly6C^high^ macrophages. Flow cytometry revealed that cCATH-3 treatment selectively enriched the Ly6C^high^ pro-inflammatory macrophage subset in peritoneal lavage, whereas cCATH-1 predominantly elicited neutrophil influx (Figure 4). Therefore, monocytes and/or derived macrophages were the primary orientation, as neutrophils and macrophages are key phagocytic cells against invading pathogens in vivo [54]. Given that the Ly6C^high^ macrophage subset is primarily pro-inflammatory, whereas the Ly6C^low^ subset is associated with tissue repair [55].

Macrophage depletion using anti-CSF1R antibody abolished cCATH-3-dependent bacterial clearance (Figure 7), establishing macrophages as essential effector cells. In vitro functional assays further demonstrated that while cCATH-1- and cCATH-3-treated macrophages showed comparable phagocytic capacity toward inactivated bacteria, cCATH-3-primed macrophages exhibited superior intracellular killing of live APEC E058 (Figure 5C). Additionally, cCATH-3 demonstrated significantly stronger chemotactic activity toward peritoneal macrophages than cCATH-1 in both single and co-culture Transwell assays (Figure 6), correlating with its enhanced induction of CCL-3 and CCL-5 in vivo and in vitro (Figures 9, 10).

The molecular basis for this differential macrophage activation lies in the distinct cytokine profiles induced by cCATH-1 and cCATH-3. In vitro cytokine profiling revealed that both cathelicidins broadly activated chemokine and cytokine transcription in peritoneal macrophages in a dose-dependent manner (Figure 8A). However, cCATH-3 selectively enhanced CCL-3 and CCL-4 expression approximately two-fold compared to cCATH-1 at 10 μM (Figure 8C). The coordinate upregulation of CCL-3, CCL-4, and CCL-5 establishes a self-amplifying chemokine cascade: CCL-3 and CCL-4 stimulate macrophages to produce IL-6 and IL-1, contributing to host defense during early infection [56–58]; macrophages recruited by CCL-3 subsequently enhance CCL-5 expression after colonization [59, 60]; and CCL-5, together with CCL-7 and CCL-8, acts as a chemoattractant for blood monocytes, further promoting their recruitment into the peritoneal cavity [61–63]. CCL-6 amplifies this response by recruiting additional macrophages to the peritoneum [64]. This sequential activation provides a mechanistic explanation for the early protection observed at 4 h post-cCATH-3 treatment.

To further investigate the role of chemokine CCL-3 in the protective effects of cCATH-3 in mice, an expression strain was constructed and biologically active mCCL3 protein was purified. A rabbit polyclonal antibody against mouse CCL-3 was prepared, and its ability to neutralize mCCL3 protein both in vivo and in vitro was validated using the purified antibody. The central role of CCL3 in cCATH-3-mediated protection was confirmed through chemokine neutralization experiments. In the challenge protection experiment, neutralization of free mCCL3 protein in vivo by rabbit anti-mouse CCL-3 polyclonal antibody completely abolished cCATH-3-dependent protection (Figure 10C). These findings further demonstrate that the protective effect of cCATH-3 in mice is mediated by stimulation of macrophages to secrete mCCL3, thereby enhancing the expression of downstream molecules such as CCL-5, which recruits monocytes from the bloodstream and increases macrophage levels in the peritoneal environment [59, 60, 63]. The expression of these factors provides a certain degree of protection against subsequent APEC E058 challenge, thereby contributing to resistance against infection.

Several limitations in this study should be highlighted. The protective efficacy of cCATH-3 was evaluated in a murine model rather than chicken model, the natural host of APEC, which may affect translational relevance. And, although CCL3 was identified as a critical mediator, the upstream receptor and signaling pathway through which cCATH-3 induces CCL3 production remain unknown.

Despite these limitations, our study has several notable findings. The direct comparison of cCATH-1 and cCATH-3—two closely related orthologs with similar structures but markedly different in vitro and in vivo activities—provides a powerful experimental paradigm to differentiate direct bacteriocidal effects from immunomodulatory mechanisms. Our multi-layered approach, combining in vitro MIC and cytotoxicity assays, in vivo survival studies, flow cytometric immune profiling, macrophage functional assays, and chemokine neutralization, allows robust causal inference regarding the CCL3-macrophage axis in cCATH-3-mediated protection. Furthermore, the identification of CCL3 as a specific, non-redundant mediator offers a concrete mechanistic target for the rational design of next-generation HDP-based therapeutics.

Future studies should focus on elucidating the precise moleculars and signaling pathway through which cCATH-3 induces CCL3 in macrophages, validating cCATH-3 efficacy in chicken APEC infection models and mapping the CCL3-inducing activity to specific structural motifs of cCATH-3 through structure–function relationship studies.

In conclusion, this study demonstrates that direct bacteriocidal activity, as measured by conventional MIC assays, is insufficient to predict the in vivo protective efficacy of avian cathelicidins. cCATH-3, despite its weaker in vitro bacteriocidal activity related to cCATH-1, confers robust protection against APEC E058 challenge in mice through a mechanism involving CCL3-dependent recruitment and activation of Ly6C^high^ macrophages. These findings challenge the traditional paradigm that prioritizes direct antimicrobial potency in HDP development and highlight the importance of evaluating immunomodulatory functions in physiologically relevant contexts. The identification of the CCL3-macrophage axis as a critical determinant of cCATH-3-mediated protection provides a mechanistic foundation for the rational design of immunomodulatory HDPs as alternatives to conventional antibiotics in combating antimicrobial-resistant APEC infections.

## Supplementary Information

 **Additional file 1 Cytotoxicity of cathelicidins on peritoneal macrophages. **Cytotoxicity of cathelicidins on peritoneal macrophages determined by CCK-8 assay.

## Data Availability

The PDB accession codes 2AMN and 2HFR were used in this study. All the other data are available from the corresponding authors upon reasonable request.
