# Supplementary material for: Chicken cathelicidin-3 protected mice from avian pathogenic Escherichia coli infection mediated by triggering chemokine CCL3 pathway
Source: Vet Res. 2026 Oct 6;57:175. doi: 10.1186/s13567-026-01844-5 (PMC13640183; doi:10.1186/s13567-026-01844-5)
Supplement: Supplementary file 1 [file 13567_2026_1844_MOESM1_ESM.docx]

**Cytotoxicity of cathelicidins on peritoneal macrophages**

**Figure S1 Cytotoxicity of cathelicidins on peritoneal macrophages determined by CCK-8 assay**

The median cytotoxic concentration (CC_50_) of cCATH-1 in treated peritoneal macrophages was determined as 78.28 μg/mL (about 23.45 μM), whereas the CC_50_ of cCATH-3 was 106.75 μg/mL (about 30.1 μM) (Figure S1).

According to these findings, nearly no cytotoxic effect was observed in peritoneal macrophages exposed to concentrations below 30 μg/mL of either cathelicidins for 4 h. In this study, concentrations ≤30 μg/mL (about 10 μM) of either cathelicidins were applied, and the incubation duration with peritoneal macrophages was maintained below 2 h to ensure that cytotoxicity of cathelicidins remained minimal.

**Characterization of mCCL3 recombinant protein and mCCL3 specific antibody**

The concentration of mCCL3 recombinant protein determined by BCA assay was 2.9 mg/mL, and the purity of this sample was about 86% based on gray scanning analysis. The median effective concentration for mouse peritoneal macrophages was 1.85–8.26 ng/mL.

The titer of mCCL3 Ab was determined to be 1:102400 by ELISA, and its titer was 1:8 as determined by agar diffusion test. The titer of the Vec Ab group against mCCL3 was less than 1:200 by ELISA. The concentrations of purified mCCL3 Ab and Vec Ab were 5 mg/mL and 2 mg/mL, respectively.

**The capacity of the mCCL3 Ab to neutralize CCL3 both *in vivo* and *in vitro***

The mCCL3 Ab was used to verify its neutralizing ability *in vitro* by Transwell assay. Both 10-fold diluted and 100-fold diluted mCCL3 Ab (5 mg/mL) inhibited the chemotactic ability of mCCL3 protein (5 μg) to recruit mouse peritoneal macrophages. The number of migrating cells after neutralization was not significantly different from that of Vec Ab-treated mCCL3 protein. At dilutions greater than 1000-fold, mCCL3 Ab lost its ability to neutralize mCCL3 protein after 8 h incubation, and the number of migrating cells was not significantly different from that of the mCCL3 protein-treated group, but was significantly different from that of the mock group (Figure S2A).

*In vivo*, the CCL-3 level in mouse peritoneal macrophages collected from mice treated with mCCL3 Ab, Vec Ab, or Mock Ab was assessed by confocal microscopy. In the IFA assay using mCCL3 Ab, mCCL3 was mainly enriched on the surface of the cell membrane Figure S2B). According to semi-quantitative analysis using ZEN 3.4 software, there was no significant difference in mCCL3 levels on the surface between Vec Ab-treated and mCCL3 Ab-treated cells, while the fluorescence intensity of mCCL3 Ab-treated cells was significantly lower than that of either Vec Ab-treated or Mock Ab-treated cells (Figure S2B, S2C). These results indicate that mCCL3 Ab may neutralize free mCCL3 protein in the mouse peritoneal cavity and prevent its adsorption onto the surface of mouse peritoneal macrophages.

**Figure S2 The capacity of the mCCL3 Ab to neutralize CCL3 in both *in vivo* and *in vitro***

**A:** Chemotactic effect of mCCL3 protein (5 μg), or mCCL3 protein incubated with differently diluted mCCL3 Ab, on mouse peritoneal macrophages in the Transwell system. ***p<*0.01 represents significant differences between cells treated with mCCL3 or mCCL3 + mCCL3 Ab/Vec Ab and cells treated with Mock Ab. **B:** CCL-3 staining in mouse peritoneal macrophages collected from mice treated with mCCL3 Ab, Vec Ab, or Mock Ab assessed by confocal microscopy. **C:** Semi-quantitative fluorescence intensity analysis of confocal microscopy observations using ZEN 3.4. **p<*0.05 represents significant differences between cells treated with Vec Ab and cells treated with mCCL3 Ab.
